# HIV-Infected Individuals Do Not Present Significant Differences regarding Periodontal Status: A Systematic Review and Meta-Analysis

**DOI:** 10.1155/2024/5559610

**Published:** 2024-08-26

**Authors:** Lucas Ribeiro Teixeira, Diana Estefania Ramos Peña, Leticia Rodrigues de Castro, Márcia dos Santos, Maria da Conceição Pereira Saraiva, Fernando Chahud, Bruno Pozzetto, Alan Grupioni Lourenço, Ana Carolina Fragoso Motta

**Affiliations:** ^1^ Department of Pathology and Forensic Medicine Ribeirão Preto Medical School University of São Paulo, Ribeirão Preto, São Paulo, Brazil; ^2^ Department of Stomatology School of Dentistry University of São Paulo, São Paulo, Brazil; ^3^ Central Library of Ribeirão Preto University of São Paulo, Ribeirão Preto, São Paulo, Brazil; ^4^ Department of Pediatric Dentistry Ribeirão Preto School of Dentistry University of São Paulo, Ribeirão Preto, São Paulo, Brazil; ^5^ Team Mucosal Immunity and Pathogen Agents International Center for Infectiology Research (CIRI) INSERM U1111 University of Lyon University of Saint-Etienne, Saint-Etienne, France; ^6^ Department of Basic and Oral Biology Ribeirão Preto School of Dentistry University of São Paulo, Ribeirão Preto, São Paulo, Brazil; ^7^ Department of Stomatology Public Health and Forensic Dentistry Ribeirão Preto School of Dentistry University of São Paulo, Ribeirão Preto, São Paulo, Brazil

## Abstract

**Objective:**

To evaluate, through a systematic literature review, whether periodontal status in HIV-infected individuals is different from those non-HIV-infected.

**Materials and Methods:**

A systematic search for published observational studies within six electronic databases and grey literature was conducted, PROSPERO database number CRD42020160062. Results from studies reporting clinical periodontal parameters: probing pocket depth, bleeding on probing, clinical attachment level, plaque index, and gingival index, in HIV- and non-HIV-infected individuals were reviewed. The quality of the assessment was evaluated according to the Joanna Briggs Institute Appraise Checklist.

**Results:**

Twenty-three observational studies met the eligibility criteria and were included for analysis. The qualitative analysis indicated similarities in periodontal parameters within both groups, with no significant mean difference (MD) within both groups regarding clinical periodontal parameters; severe heterogeneity was also detected.

**Conclusions:**

No significant differences were found in the periodontal profile of HIV-infected and non-HIV-infected individuals. However, the high heterogeneity among the studies calls for caution in interpreting these findings. Further investigations using standardized methods for periodontal evaluation are needed to clarify the association between HIV infection and periodontal conditions.

## 1. Introduction

The human immunodeficiency virus type 1 (HIV-1) infection is characterized by systemic immunosuppression, associated with depletion of cluster of differentiation (CD) 4 positive T-cells (CD4+ T-cells) that can lead to acquired immunodeficiency syndrome (AIDS), causing important clinical manifestations [1]. The development of combination antiretroviral therapy (cART) has led to the control of viral replication and decrease of HIV/AIDS-related illnesses, reducing the morbidity and mortality, influencing positively the improvement of overall quality of life, and consequently a near-normal life expectancy of persons living with HIV (PLHIV) [2]. The World Health Organization (WHO) has estimated 39 million PLHIV and approximately 630,000 deaths from HIV-related diseases in 2022, mainly in developing areas of Africa, Asia, and Americas [3]. Among the multisystemic manifestations of HIV/AIDS, those affecting oral structures have been associated with markers of immune dysfunction, being strongly associated with oral candidiasis (OC), hairy leukoplakia, and an increased risk of malignant neoplasms such as Kaposi sarcoma (KS) and non-Hodgkin lymphoma (NHL) [4]. Linear gingival erythema (LGE), necrotizing gingivitis (NG), and necrotizing periodontitis (NP) have been also associated with the HIV/AIDS infection [5, 6, 7]. Besides these specific forms of periodontal diseases, periodontitis was also shown to occur and to progress differently in this population as a result of immunosuppression. In the cART era, a significant decrease in the incidence of oral AIDS-related illnesses has been observed; however, the role of cART on the frequency and severity of periodontitis (PD), the most common oral disease in the general population, remains poorly understood in this group [6, 8, 9].

Periodontal condition is influenced by environmental (e.g., smoking), psychological, and immunological factors. In PLHIV, these variables can differ from the general population [10, 11, 12], raising questions about the clinical and microbiological status of the periodontium in this condition. Primary studies have investigated the influence of HIV on clinical periodontal parameters; however, no systematized research has been conducted on the periodontal status between individuals with and without HIV infection. Therefore, the purpose of this systematic review was to determine within observational reports whether there are differences in the periodontal conditions of HIV-infected and non-HIV-infected individuals.

## 2. Materials and Methods

### 2.1. Protocol and Registration

This study was conducted in accordance with the preferred reporting items for systematic reviews and meta-analyses (PRISMA) updated guideline [13]. The review protocol was registered in the PROSPERO database, under the protocol number CRD42020160062.

### 2.2. Eligibility Criteria Based on PECO Framework and Review Question

The research question of this systematic review was “Is periodontal condition of HIV-infected individuals different from that of non-HIV-infected individuals?” The inclusion criteria followed the PECO framework, being P (population): human adults assessed for periodontal parameters; E (exposure): human immunodeficiency virus (HIV-1) infection; C (comparison): non-HIV-infected adults; and O (outcome): For quantitative results, periodontal status assessed by clinical parameters mean probing pocket depth (PPD), mean bleeding on probing (BOP), mean clinical attachment level (CAL), mean plaque index (PI), and mean gingival index (GI). For qualitative results, main conclusions and tendencies described by the authors regarding the periodontal status and/or clinical periodontal parameters.

### 2.3. Inclusion and Exclusion Criteria

Observational clinical studies in adult human patients (including, cohort, case-control, and cross-sectional designs with ethical approval) that comparatively assessed at least one clinical periodontal parameter in HIV-infected versus non-HIV-infected individuals were included. Exclusion criteria includes studies performed in animal models or “*in vitro*” tests; studies that do not include a non-HIV-infected control group; studies assessing pediatric, pregnant, or diabetic patients.

To avoid duplication of data collection and entry, situations in which similar authorship, sample size, or source of patient recruitment was found, the corresponding author was contacted to verify for repeated data [14, 15]. When this information could not be obtained, the study with the largest sample size was included. Importantly, studies performed on the same sample but analyzing different outcomes were also included.

### 2.4. Literature Search Strategy

A comprehensive search strategy was performed by one of the authors (L.R.T.) and an experienced librarian (M.S.), on the electronic databases: PubMed, Web of Science, Scopus, Embase, LILACS (Latin American and Caribbean Health Sciences Literature), and DOSS (Dentistry & Oral Sciences Source), and grey literature through Google Search engine and Google Scholar, without restriction of publication date or language (last search: July 2023). The search strategy was used for each electronic database using appropriate controlled vocabulary (Medical Subject Heading Terms—MeSH for PubMed; Emtree in Embase; *Descritores em Ciências da Saúde—*DeCS for LILACS) and free terms combined by the Boolean operators *“AND”* and *“OR”* (*Supplementary 1*). In addition, a manual search was performed through the reference lists of all selected studies for relevant references.

### 2.5. Study Selection and Data Extraction

The studies retrieved from the literature search were uploaded into RAYYAN QCRI web application [16] for identification and removal of duplicates. Study selection was conducted by two reviewers (L.R.T. and L.R.C.) independently in two steps. First, the studies were screened by title and abstracts, and second, a full-text review for the final study selection was conducted. Disagreements were consulted with a third reviewer (A.C.F.M.) and resolved after discussion and consensus.

Outcome data were extracted from the included studies by two reviewers (L.R.T. and D.E.R.P.) and cross-checked by a third one (A.C.F.M) using a customized form for assessment of qualitative and quantitative data including basic information (authors, country, year of publication); study design; sample characteristics (sample size of HIV-infected and non-HIV-infected individuals), mean age, gender, laboratory tests (plasma HIV viral load and CD4 counts), cART status (on cART or not); periodontal clinical assessment including PPD, BOP, CAL, PI, and GI.

### 2.6. Quality of Assessment (QoA) in Individual Studies

The methodological quality of each study was analyzed independently by two reviewers (L.R.T. and D.E.R.P.) according to the Joanna Briggs Institute's Critical Appraisal Checklist for observational studies, which includes questions according to study design that could be answered with “yes,” “no,” “unclear,” or “not applicable.” The QoA of individual studies was categorized as high-QoA (49% or less “yes” answers), moderate QoA (50%–69% “yes” answers), and low-QoA (70% or more “yes” answers). Disagreements or uncertainties were resolved by a third reviewer (A.C.F.M.).

### 2.7. Heterogeneity and Publication Bias

Heterogeneity of each periodontal parameter was quantified by I1 statistics, a value of 50% indicated substantial heterogeneity. A funnel plot was constructed to assess publication bias.

### 2.8. Statistical Analysis

For continuous data (PPD, BOP, CAL, PI, and GI), mean, standard deviation (SD), and 95% confidence interval (CI) were calculated for the studies that presented these data, using the inverse variance statistical method. The Review Manager Software (RevMan) [17] was used for quantitative analysis and to generate the figures.

## 3. Results

### 3.1. Search Results

**Figure 1:**
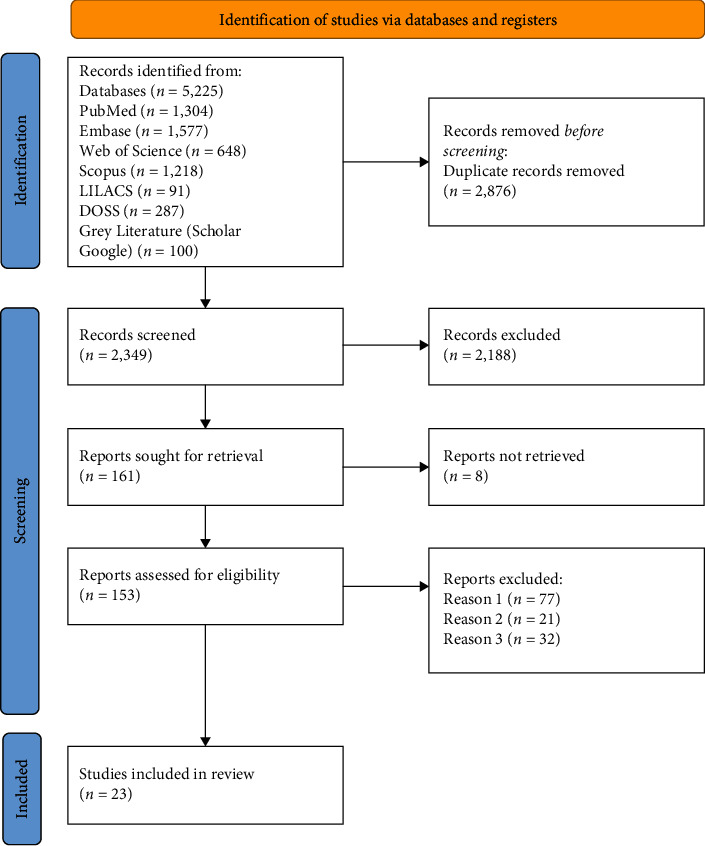
Study selection process according to PRISMA 2020 flow diagram.

The search identified 5,225 studies in electronic databases, after the removal of duplicates, 2,349 articles were screened by title and abstract, and 153 articles were full-text screened, out of these, 23 articles that met the eligibility criteria were included (Figure 1), no additional relevant articles were found through manual search of the reference lists. A detailed summary of the excluded studies is provided in *Supplementary 2*.

### 3.2. General Characteristics of the Selected Studies

Among the study design of the included studies, 21 articles were cross-sectional and two cohorts. The selected studies were carried out in 11 countries, Brazil (*n* = 7), United States (*n* = 5), United Kingdom (*n* = 3), South Africa (*n* = 1), Croatia (*n* = 1), Tanzania (*n* = 1), Senegal (*n* = 1), Venezuela (*n* = 1), Colombia (*n* = 1), Sweden (*n* = 1), and Thailand (*n* = 1). The oldest paper was published by Lucht et al. [18], and the most recent was published by Pereira et al. [19]. Three studies were published in pre-cART era (before 1996), and 20 studies were published in the cART era (from 1996 onwards), no ethical issues were detected among the studies. The total sample size ranged from 32 to 735 individuals [20, 21]. In HIV-infected and non-HIV-infected individuals, the smallest sample size was 11 [22] and 10 [18, 21], and the largest one was 584 [20, 23] and 406 [24], respectively. The reported number of HIV women was higher than that of men (1,649 versus 1,094). Three reports included only women [20, 23, 25], whereas one study assessed only men [26]. A summary of the demographic and serological characteristics of the included studies is reported in Table 1.

### 3.3. Quality of Assessment

**Figure 2:**
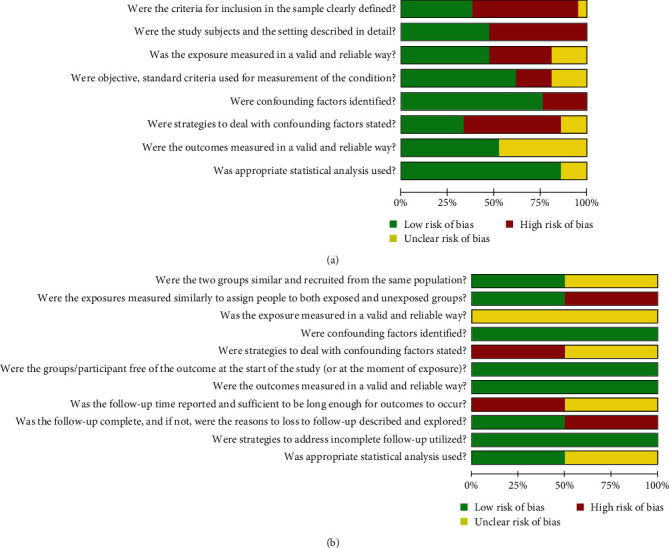
Quality of assessment graph: review authorsʼ judgements presented as percentages across of cross-sectional (a) and cohort studies (b), analyzed according to the Joanna Brigs Institute (JBI) checklist.

Among the cross-sectional studies, most studies (14/21, 61.9%) were classified as moderate QoA [21, 22, 23, 24, 25, 26, 27, 29, 31, 32, 33, 34, 36, 38]. Four studies (19.05%) were classified as low QoA [18, 28, 35, 39], and three (19.05%) were classified as high QoA [19, 37, 40]. The main points that decreased the QoA were related to definitions of inclusion criteria, description of the study subject, and strategies of confounding factors. Regarding cohort studies, one study was considered as high QoA [20], and one as low QoA [30] due to unclear exposure measurements and strategies for confounding factors. (Figure 2 and *Supplementary 3*).

### 3.4. Sensitivity Analysis, Heterogeneity, and Publication Bias

**Figure 3:**
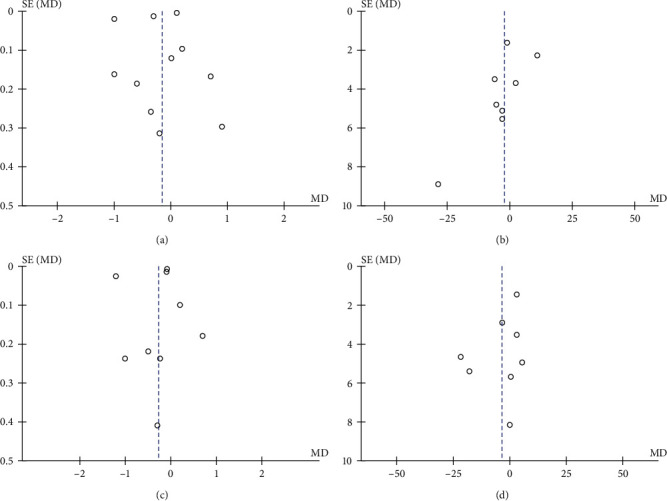
Heterogeneity of studies included in the quantitative analysis. Funnel plot for probing pocket depth (a), bleeding on probing (b), clinical attachment level (c), and plaque index (d).

The most commonly reported periodontal parameter in the included studies was PPD (*n* = 18), followed by BOP (*n* = 13), CAL (*n* = 13), PI (*n* = 14), and GI (*n* = 4); due to the inconsistency of the reporting methods, sensitivity analysis was performed only in 11 studies for PPD, eight studies for BOP, nine studies for CAL, and eight studies for PI. We were not able to perform sensitive analysis for GI due to low data and discrepancies in the presentation of this parameter. High heterogeneity was found among the studies PPD (*χ*^2^ : 3,388.0, *p*  < 0.00001, *I*^2^ : 100%), BOP (*χ*^2^ : 38.28, *p*=0.00001, *I*^2^ : 82%), CAL (*χ*^2^: 1,736.01, *p*  < 0.00001, *I*^2^: 100%), and PI (*χ*^2^: 40.43, *p*  < 0.00001, *I*^2^: 83%). The funnel plot also showed asymmetry for the five periodontal parameters (Figure 3).

### 3.5. Immunological Assessment of HIV-Infected Individuals

Respecting the immunological status of the HIV-infected individuals, 12 studies reported viral load, 19 studies reported CD4 counts, and 16 studies reported cART status (Table 1). Three studies included only HIV-infected individuals with undetectable or <50 copies/mm^3^ [19, 22, 38], four studies included individuals with detectable viral load [29, 32, 33, 35], and five studies included HIV-infected individuals with both detectable and undetectable viral load [21, 23, 34, 36, 40].

### 3.6. Periodontal Parameters

Data obtained for periodontal parameters, including PPD, CAL, BOP, PI, GI, and periodontal status are summarized in Table 2 and described in the following sections.

### 3.7. Probing Pocket Depth (PPD)

**Figure 4:**
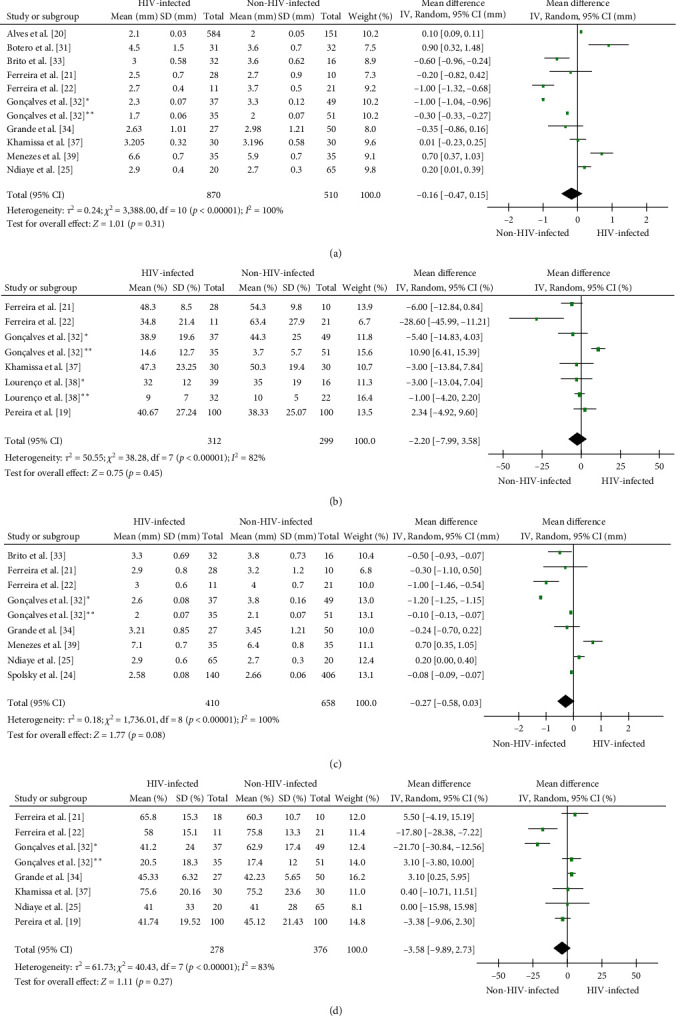
Forest plot of comparison between HIV-infected and non-HIV-infected individuals according to periodontal parameters. Probing pocket depth (a), bleeding on probing (b), clinical attachment level (c), and plaque index (d).  ^*∗*^Individuals with periodontitis and  ^*∗∗*^Individuals with periodontal health.

PPD was reported in 18 studies, being reported as mean PPD in 13 studies, mean of sites ≥4 mm/≥5 mm in two, and number of individuals with PPD ≥4 mm in two studies. Considering the different classifications of the obtained results of PPD, in six studies, HIV-infected individuals presented higher mean measurements of PPD than non-HIV-infected counterparts [18, 25, 26, 31, 36, 39], whereas five different studies exhibited the opposite pattern [21, 22, 32, 33, 34]; seven studies showed no significant differences between both groups [20, 27, 29, 35, 37, 38, 40]. Furthermore, two studies showed different results according to each group of study, Grbic et al. [27] reported that HIV-infected individuals presented higher number of sites ≥4 mm than non-HIV individuals; however, HIV parenteral drug users presented lower number of sites ≥4 mm than non-HIV-infected parenteral drug users (34.0 versus 36.7, respectively). Nittayananta et al. [35] described three groups among the HIV-infected individuals, the number of individuals without cART treatment with PPD ≥4 mm was higher in comparison to the non-infected individuals, although short-term cART presented lower number of individuals with PPD ≥4 mm and long-term cART did not present differences. On the other hand, Lucht et al. [18] observed that AIDS-related complex (ARC) and AIDS individuals had more PDD ≥4 mm compared to stable HIV-infected and non-HIV-infected individuals. The quantitative analysis of 11 studies showed (mean difference: −0.16; 95% CI: −0.47, 0.15) no differences in PPD measures between HIV-infected and non-HIV-infected individuals (Figure 4(a)).

### 3.8. Bleeding on Probing (BOP)

BOP was reported by 13 studies, being described as mean percentage of BOP by eight studies, number of sites with BOP by two studies, number of individuals with the presence of BOP by two studies, and median of sites with BOP by one study. Six studies did not detect significant differences in the occurrence of BOP between HIV-infected and non-HIV-infected individuals [19, 28, 29, 35, 37, 38]. Three studies reported a higher percentage of BOP in HIV-infected individuals in comparison to non-HIV-infected individuals [26, 31, 40], whereas four different studies showed the opposite pattern [21, 22, 27, 32]. Gonçalves et al. [32] observed that HIV-infected individuals with PD presented lower percentage of BOP compared to non-HIV-individuals with PD; however, in periodontal health (PH), HIV-infected individuals had higher BOP than non-HIV-individuals. Ferreira et al. [22] reported a significant difference regarding BOP in non-HIV-infected individuals (*p*=0.006) compared to HIV-infected individuals (34.8% versus 63.4%). The quantitative analysis of the eight analyzed studies showed a similar percentage of BOP (mean difference: −2.20; 95% CI: −7.99, 3.58) in HIV-infected and non-HIV-infected individuals (Figure 4(b)).

### 3.9. Clinical Attachment Level (CAL)

Fourteen studies reported CAL results, 10 studies reported CAL as mean, two studies as number of individuals with CAL ≥3 mm or ≥4 mm, and two studies as number of sites with CAL ≥3 mm or ≥4 mm. Four studies showed no significant differences between HIV-infected and non-HIV-infected individuals [24, 28, 29, 33]. Six studies reported higher CAL [20, 25, 26, 30, 36, 39], and four others lower CAL [21, 22, 32, 34] in HIV-infected individuals by comparison to non-HIV-infected individuals. Quantitative analysis of nine studies showed similar CAL measures (mean difference: −0.17; 95% CI: −0.64, 0.31) between HIV-infected and non-HIV-infected individuals (Figure 4(c)).

### 3.10. Plaque Index (PI)

PI was reported by 14 studies, being reported as mean percentage in 10 studies, median by one study, mean proportion of surfaces in one study, number of individuals with plaque in one study, and number of individuals with PI >10% in one study. PI was the periodontal parameter that presented less variation between both HIV-infected and non-HIV-infected individuals, with 10 studies reporting PI scores without significative difference [18, 19, 23, 25, 28, 30, 33, 34, 37, 40]. Stojković et al. [36] and Ferreira et al. [21] observed higher PI in HIV-infected individuals than in noninfected individuals, whereas two other studies reported the opposite pattern [22, 32]. Gonçalves et al. [32] also observed that a higher PI was associated with PH in HIV-infected individuals, when compared to non-HIV-infected individuals. Lucht et al. [18] verified that ARC and HIV-infected individuals had higher PI scores in comparison to AIDS individuals with PD or with noninfected individuals. The quantitative analysis of eight studies showed similar PI percentages (mean difference: −3.71; 95% CI: −11.40, 3.98) between HIV-infected and non-HIV-infected individuals (Figure 4(d)).

### 3.11. Gingival Index (GI)

GI was reported in four studies [25, 26, 31, 33]. GI was reported as mean score by three studies [26, 31, 33] and as mean percentage of sites with scores >1 by one study [25]. Robinson et al. [26] reported significative difference between GI scores, with HIV-infected patients presenting more frequency of gingival inflammation. No significative difference was reported by the other studies. A quantitative assessment could not be performed due to discrepancies in the presentation of the results.

### 3.12. Periodontal Status

In the 23 studies analyzed, the periodontal diagnosis of the participants was disclosed in 13 studies; however, most of the studies had paired the study population, either by periodontal status or by number of individuals in each group of study. We were not able to perform quantitative assessment on the prevalence of periodontitis in HIV-infected individuals due to this pairing performed by the included studies. Only two studies did not perform the selection of patients based on the periodontal status as an inclusion criteria [25, 26]; in both studies, the frequency of PD was higher in HIV-infected individuals. Among the included studies, a total of 497 individuals presented a diagnosis of periodontitis, of these 296 were HIV-infected and 201 non-HIV-infected individuals.

## 4. Discussion

In the current cART era, the frequency of intraoral lesions observed in HIV individuals has changed. Strongly HIV/AIDS-associated lesions, such as oral hairy leukoplakia, KS, GN, PN, and LGE incidence have significantly decreased. However, other lesions like human papilloma virus (HPV), caries, and periodontitis seem to be increased in HIV-infected individuals [41], thus oral healthcare needs to be updated and adequate to the current necessities of this population. Despite substantial data regarding the relationship between the HIV-immunopathophysiology and periodontal tissues, limited literature presenting a comparative analysis of periodontal clinical parameters in HIV-infected and non-HIV-infected individuals is available. Based on our knowledge, this systematic review is the first to appraise periodontal parameters according to HIV serostatus.

We gathered data regarding the current periodontal parameters described by the last workshop classification system for periodontal diseases [42]. In a general view, the quantitative analysis results of our systematic review and meta-analysis suggest comparable periodontal patterns in HIV-infected and non-HIV-infected individuals, with no statistical differences regarding PPD, BOP, CAL, or PI. A possible explanation for this is directly related to development and improvement of cART and its availability of control HIV replication and immunosuppression-related manifestations [9]. Also, the improved access to healthcare and socioeconomic conditions of HIV-infected individuals has improved in the last decades [6, 43]. Indeed, most of the studies (20/23) included in this review were published in the cART era. Nevertheless, the similarity in periodontal profile between HIV-infected and non-HIV-infected individuals had already been noticed in the pre-cART era: in a cross-sectional study, Scheutz et al. [29] reported no significant difference in PPD and BOP between both groups, suggesting that both frequency and severity of PD in HIV-infected individuals could be lower than hypothesized. Moreover, some studies on PD have highlighted the influence of other variables such as smoking, oral hygiene, age, and genetic susceptibility, rather than HIV infection [28, 29, 44, 45]. Furthermore, in a different study that evaluated periodontal status in HIV-infected individuals, the cause of poorer periodontal condition compared to uninfected ones was mainly explained by poor oral hygiene [36].

On the other hand, some reports have described that non-HIV-infected individuals present worse periodontal conditions than HIV-infected individuals. Gonçalves et al. [32] discussed that the lower levels of periodontal destruction and inflammation in HIV-infected patients with PD could be correlated with to a reduced levels of periodontal pathogenic microorganisms compared to non-HIV-infected individuals with PD, as result of the protective effect of cART in the subgingival microbiota. Interestingly, Brito et al. [33] observed deeper PPD, but no difference in CAL, PI, or GI as well as higher frequency of *Prevotella intermedia*, *Porphyromonas gingivalis*, and *Aggregatibacter actinomycetemcomitans* in non-HIV-infected individuals than in HIV-infected individuals; however, these pathogens were less frequent in HIV-infected individuals who were not under cART as compared to cART-treated ones, indicating that cART would have no effect on the subgingival population. Nonetheless, Perreira et al. [19] and Gonçalves et al. [40] found a different correlation, where HIV-infected patients with cART presented higher BOP, being twice as likely to have PD than noninfected individuals. The HIV-infected individuals' worse periodontal status despite a lower frequency of classical periodontopathogenic bacteria have been associated with dysbiosis of the subgingival biofilm and were normally commensal and health-related bacterial could lead to higher PPD and BOP [46, 47].

From a pathophysiological point of view, Alves et al. [20] discussed that the influence of cART on PD can be difficult to interpret alone and should be considered in conjunction with other important variables such as CD4 counts and viral load. In fact, the interplay of immunological and virological status on the periodontal profile has also been extensively investigated, with conflicting results [6, 9, 43]. The comparison of these laboratory markers in HIV-infected and non-HIV-infected individuals using statistical tools is scarce in the literature. In the course of this review, only three studies [18, 29, 40] included separated PPD parameters for HIV and AIDS patients, of these, only one study [18] reported differences in PPD, with an increase in PPD in AIDS; however, the PPD cut-point used in this study was ≥4 mm for periodontitis, currently is considered ≥5 mm. Additionally, one report compared periodontal parameters (PPD, CAL, PI, and GI) in subgroups of HIV-infected (CD4 counts > 200 cells/mm^3^ and <200 cells/mm^3^) and non-HIV-infected individuals, although with no significant differences between the groups [33].

The effect of cART on the severity of the periodontal status was not an objective of this review; however this topic was directly approached by the studies of Pereira et al. [19], Nittayananta et al. [35], Gonçalves et al. [40], and Khammissa et al. [37]. While Gonçalves et al. [40] and Khammissa et al. [37] described positive effects of cART on the periodontal status of the participants, Pereira et al. [19] found a correlation with long-term use of non-nucleotide reverse transcriptase inhibitors (NNRTIs) and the development of periodontitis. Nittayananta et al. [35] also described a negative effect on the periodontal status after the use of long-term cART.

These results indicate, from a clinical point, that the periodontal management of HIV-infected individuals should not differ from that of non-HIV-infected ones, including regular monitoring of clinical conditions (dental plaque and bleeding control) and professional procedures (scaling and root planing). Importantly, a study observed an increase in CD4 counts and a reduction in viral load after nonsurgical periodontal therapy in HIV-infected individuals with PD [48], illustrating the benefits of periodontal treatment in immunological parameters. In addition, compliance with home care orientations and tobacco cessation is essential for PH maintenance [41]. Although the effect of smoking in the periodontal parameters was not a focus of this review, an important percentage of smokers was observed in the HIV-infected individuals of the included studies that reported these data [19, 20, 21, 23, 24, 28, 30, 32, 34, 35, 36, 37, 38, 39, 40]. These considerations should be shared with healthcare providers and patients to raise awareness regarding the link between HIV infection and periodontitis development.

The results of this systematic review give a global overview of the periodontal pattern according to HIV status, but must be interpreted cautiously, due to substantial heterogeneity among studies regarding differences in populations, cART modalities, and assessment of periodontal parameters. For example, the studies included in this review were developed in different geographic regions, including high-income (e.g., US and UK) and middle- to low-income countries (e.g., Brazil, India, and South Africa), different age groups (young adults, adults, and elderly), or including specific groups, such as homosexual men [26], female sex workers [25], or parenteral drug users [27], contributing to the heterogeneity of populations. Additionally, several discrepancies in the method of assessment and report of the periodontal parameters were identified, limiting the determination of the quantitative overview. For example, PPD is the most commonly used parameter to define PH [49]. However, the methods of measurement and cut-off values for the periodontal parameters varied considerably among the articles included in this review. These variations hindered further analysis, such as assessing the severity of periodontal status in relation to HIV status. These discrepancies were reduced in the more recent studies, highlighting the importance of standardized and actualized classification systems, such as those proposed by the American Academy of Periodontology and the European Federation of Periodontology [42]. Lastly, inferring the effect of cART on periodontal status would introduce significant bias. This is because most studies reporting cART did not specify the prescribed regimens, nor did they correlate periodontal parameters with the type, duration or resistance of cART, except for the study of Pereira et al. [19]. These limitations stemming from heterogeneity restrict the feasibility of conducting a comprehensive meta-analysis and drawing more objective conclusions.

To conclude, this study only focuses on the assessment of periodontal parameters since the estimation of the prevalence of periodontitis in HIV-infected patients is not possible from a practical point of view. In the included studies, and even in those that did not reach the inclusion criteria, the prevalence of periodontitis was determined by each author, impossibiliting the estimate of a more complex epidemiological approach. Additionally, the updates in the periodontal classification in the last decade would interfere with the diagnosis of periodontitis in the earlier studies, since different criteria and parameters have been used during the development of periodontal studies. This also applies to cART effects on periodontal status severity, since several studies were developed at the beginning of the cART era, more longitudinal data are needed to describe the effects of cART on periodontal findings.

## 5. Conclusions

Based on the studies included in this systematic review, no significant differences were found regarding periodontal parameters (PPD, BOP, CAL, PI, and GI) between HIV-infected and non-HIV-infected individuals, suggesting a similar periodontal status between both groups. However, the considerable heterogeneity among the studies reinforces the need for further studies with high reproducibility and standardized methods of measurement and presentation of the results, in view of a better characterization of the periodontal status of HIV-infected individuals and its implication in the management of periodontal disease for this at-risk population.

## Figures and Tables

**Table 1:** Study data, demographic, and clinical characteristics of individuals of the 23 selected studies, classified by chronological order comparing HIV-positive and HIV-negative subjects.

Authors (year)	Country^1^	Study design	HIV-infected individuals				Non-HIV-infected individuals	
			Sample size (%)	Age (%)	Serological characteristics	Antiretroviral therapy status (%)	Sample size (%)	Age (%)
Lucht et al. [18] (1991)	Sweden	Cross-sectional	*N* = 30 Male: *N* = 28 (93.33) Female: *N* = 2 (6.67)	Individuals without clinical signs of infection: mean 35.5 years ARC individuals: mean 42.3 years AIDS individuals: mean 38.1 years	HIV viral load (mean): NA CD4 count: Individuals without clinical signs of infection: 0.53 × 10^6^/L ARC individuals: 0.24 × 10^6^/L AIDS individuals: 0.06 × 10^6^/L	NA	*N* = 10 Male: *N* = 10 (100) Female: *N* = 0	Mean: 37.7 years

Grbic et al. [27] (1995)	United States of America	Cross-sectional	*N* = 121 Male: *N* = 105 (86.77) Female: *N* = 16 (13.23)	HIV-infected homosexual men (mean): 40.9 years HIV-infected parenteral drug users (mean): 39.3 years	HIV viral load: NA CD4 count (mean): HIV-infected homosexual men individuals: 305 cells/mm^3^ HIV-infected parenteral drug users: 402 cells/mm^3^	On ART: Homosexual men: 48.6% HIV-infected parenteral drug users: 63.0%	*N* = 83 Male: *N* = 70 (84.3) Female: *N* = 30 (15.7)	Homosexual men without HIV: mean 40.0 years Parenteral drug users: mean 41.6 years

Smith et al. [28] (1995)	United Kingdom	Cross-sectional	*N* = 29 Male: *N* = 23 (79.3) Female: *N* = 6 (20.7)	Mean: 33.2 years	HIV viral load: NA CD4 count: NA	NA	*N* = 27 Male: *N* = 18 (66.6) Female: *N* = 9 (33.3)	Mean: 35 years

Robinson et al. [26] (1996)	United Kingdom	Cross-sectional	*N* = 312 Male: *N* = 312 (100) Female: *N* = 0	Mean: 35.9 years	HIV viral load: NA CD4 count: mean 325 cells/mm^3^	NA	*N* = 260 Male: *N* = 260 (100) Female: *N* = 0	Mean: 31.2 years

Ndiaye et al. [25] (1997)	Senegal	Cross-sectional	N = 20 Male: *N* = 0 Female: *N* = 20 (100)	Mean: 34 years	HIV viral load: NA CD4 count (mean): 620 × 10^6^/L (range: 102–1.038)	Without ART: *N* = 20 (100)	*N* = 65 Male: *N* = 0 Female: *N* = 65 (100)	Mean: 33 years

Scheutz et al. [29] (1997)	Tanzania	Cross-sectional	N = 191 Male: *N* = 62 (32.4) Female: *N* = 129 (67.5)	Mean age: 35.7 years	HIV viral load: NA CD4 count (mean): Individuals with HIV >500 cells/mm^3^: 20 20–500 cells/mm^3^: 47 <200 cells/mm^3^: 52 AIDS individuals >500 cells/mm^3^: 7 20–500 cells/mm^3^: 69 <200 cells/mm^3^: 96	Without ART: *N* = 191 (100)	*N* = 156 Male: *N* = 136 (87.2) Female: *N* = 20 (12.8)	Mean: 28.3 years

Robinson et al. [30] (2000)	United Kingdom	Cohort	*N* = 19 Male: *N* = 18 (94.74) Female: *N* = 1 (5.26)	Mean: 33.3 years	HIV viral load: NA CD4 count (mean): 339 cells/mm^3^	NA	*N* = 19 Male: *N* = 17 (89.5) Female: *N* = 2 (10.5)	Mean: 34.3 years

Mulligan et al. [23] (2004)	United States of America	Cross-sectional	*N* = 584 Male: *N* = 0 Female: *N* = 584 (100)	Mean: 37 years	HIV viral load: ≤4,000 cp/mL: *N* = 173 (31) 4,001–50,000 cp/mL: *N* = 200 (35) >50,000 cp/mL: *N* = 193 (34) CD4 count: ≤200: *N* = 151 (27) 201−500: *N* = 238 (42) >500: *N* = 175 (31) Unknown *N* = 18	On ART: *N* = 353	*N* = 151 Male: *N* = 0 Female: *N* = 151 (100)	Mean: 36 years

Alves et al. [20] (2006)	United States of America	Cohort	*N* = 584 Male: *N* = 0 (0) Female: *N* = 584 (100)	NA	HIV viral load: NA CD4 count: NA	NA	*N* = 151 Male: *N* = 0 (0) Female: *N* = 151 (100)	NA

Botero et al. [31] (2007)	Colombia	Cross-sectional	*N* = 31 Male: *N* = 26 (89.9) Female: *N* = 5 (16.1)	Mean: 37.3 years	HIV viral load: NA CD4 count: NA	On ART: *N* = 22 (70.97)	*N* = 64 Male: *N* = 11 (34.4%) Female: *N* = 21 (65.6)	Mean: 40.8 years

Gonçalves et al. [32] (2007)	Brazil	Cross-sectional	*N* = 72 Male: *N* = 47 Female: *N* = 25	Mean: Individuals with CP: 40.0 years Individuals with PH: 37.0 years	HIV viral load: Individuals with CP: 33,071 ± 92,139 cp/mm^3^ Individuals with PH: 23,960 ± 65,673 cp/mm^3^ CD4 count: HIV + individuals with PD: 382 ± 268 cells/mm^3^ HIV + individuals with PH: 333 ± 195 cells/mm^3^	On ART: *N* = 72 (100)	*N* = 100 Male: *N* = 51 Female: *N* = 49	Mean: Individuals with CP: 43.4 years HIV-individuals with PH: 34.4 years

Brito et al. [33] (2008)	Venezuela	Cross-sectional	*N* = 32 Male: *N* = 27 (84.37) Female: *N* = 5 (15.63)	Mean: 37.09	HIV viral load (mean): Individuals on ART: 68,850 ± 184.725 cps/mm^3^ Individuals without ART: 170,190 ± 189,934 cps/mm^3^ CD4 count: <200 cells/mm^3^: *N* = 11 (34.3) >200 cells/mm^3^: *N* = 21 (65.6)	On ART: *N* = 21 (21.62)	*N* = 16 Male: *N* = 5 (31.3) Female: *N* = 11 (68.7)	Mean: 41.62 years

Grande et al. [34] (2008)	Brazil	Cross-sectional	*N* = 50 Male: *N* = 35 (70) Female: *N* = 15 (30)	Mean: 41.2 years	HIV viral load: undetectable: *N* = 29 (58) <10,000 cp/mL: *N* = 14 (28) >10,000 cp/mL: *N* = 7 (14) CD4 count: <200 cells/mm^3^: *N* = 5 (10) 200–499 cells/mm^3^: *N* = 23 (46) >500 cells/mm^3^: *N* = 21 (42) Not determined: *N* = 1 (2)	On ART: *N* = 40 (80)	*N* = 50 Male: *N* = 28 (56%) Female: 22 (44%)	Mean: 41.74 years

Nittayananta et al. [35] (2010)	Thailand	Cross-sectional	*N* = 157 Male: *N* = 71 (34.29) Female: *N* = 86 (65.71)	Mean: Individuals with short-term ART: 37 years Individuals with long-term ART: 40 years Individuals without ART: 34 years	HIV viral load (mean): individuals with short-term ART: 21.560 cp/mL Individuals with long-term ART: 5,627 cp/mL Individuals without ART: 782 cp/mL CD4 count: individuals with short-term ART: 250 cells/mm^3^ individuals with long-term ART: 531 cells/mm^3^ individuals without ART: 245 cells/mm^3^	On short-term ART: *N* = 45 On long-term ART: *N* = 54 Without ART: *N* = 58	*N* = 50 Male: *N* = 25 (50) Female: *N* = 25 (50)	Mean: 36 years

Stojkovic et al. [36] (2011)	Croatia	Cross-sectional	*N* = 25 Male: *N* = 19 (76) Female: *N* = 6 (24)	Mean: 40.8 years	HIV viral load: ranging from below 20–1,650,000 cp/mm^3^ CD4 count (median): 165 cells/mm^3^	NA	*N* = 25 Male: *N* = 18 (72) Female: *N* = 7 (28)	Mean: 40.92 years

Khamissa et al. [37] (2012)	South Africa	Cross-sectional	*N* = 30 Male: *N* = 10 (33) Female: *N* = 20 (67)	18–45 years	HIV viral load: NA CD4 count: Individuals in ART: 172 cells/mm^3^ ART-naïve individuals: 257 cells/mm^3^	On ART: *N* = 16 (53.33)	*N* = 30 Male: *N* = 16 (53.3) Female: *N* = 14 (66.7)	18–45 years

Lourenço et al. [38] (2013)	Brazil	Cross-sectional	*N* = 71 Male: *N* = 30 Female: *N* = 41	Mean: Individuals with PH: *N* = 40 years Individuals with CP: *N* = 39 years	HIV viral load: <50 copies: *N* = 71 (100) CD4 count: Individuals with PH: 476 cell/mm^3^ Individuals with CP: 476 cell/mm^3^	On ART: individuals with PH: *N* = 26 (81) individuals with CP: *N* = 32 (82)	*N* = 38 Male: *N* = 19 Female: *N* = 19	Mean: Individuals with PH: 33 years Individuals with CP: 38 years

Ferreira et al. [21] (2015)	United States of America	Cross-sectional	*N* = 28 Male: *N* = 21 (75) Female: *N* = 7 (25)	Median: 41 years	HIV viral load: ranging from 0 to 510,242 cp/mm^3^ CD4 count (mean): 430 ± 323 cells/mm^3^	Without ART: 28 (100)	*N* = 10 Male: *N* = 6 (60) Female: *N* = 4 (40)	Median: 47.5 years

Ferreira et al. [22] (2016)	Brazil	Cross-sectional	*N* = 11 Male: *N* = 7 (63.6) Female: *N* = 4 (36.4)	Mean: 47.3 years	HIV viral load: undetectable: *N* = 11 (100) CD4 count (mean): 618 cells/mm^3^	On ART: *N* = 11 (100%)	*N* = 21 Male: *N* = 10 (47.6) Female: *N* = 11 (52.4)	Mean: 45 years

Menezes et al. [39] (2018)	Brazil	Cross-sectional	*N* = 35 Male: *N* = 20 (58.4%) Female: *N* = 15 (41.6%)	Mean: 47.1 years	HIV viral load: NA CD4 count: NA	On ART: *N* = 35 (100)	*N* = 35 Male: *N* = 18 (52.9) Female: *N* = 17 (47.1)	Mean: 47 years

Spolsky et al. [24] (2018)	United States of America	Cross-sectional	*N* = 140 Male: *N* = 133 (95) Female: *N* = 7 (5)	Mean: 45.5 years	HIV viral load: > 50 cp/mm^3^: 38% CD4 count: <20 cells/mm^3^: 12%	On ART: *N* = 140 (100).	*N* = 406 Male: *N* = 308 (75.9) Female: *N* = 98 (24.1)	Mean: 43.8 years

Gonçalves et al. [40] (2022)	Brazil	Cross-sectional	*N* = 74 Male = 48 (64.9) Female = 26 (35.1)	18–35 years: *N* = 25 (34.3) 36–50 years: *N* = 41 (56.2) >50 years: *N* = 7 (9.6)	HIV viral load (cp/mL): <1,000: 21 (58.3) 1,001−10,000: 7 (19.5) >10,000: 8 (22.5) CD4 count: <200: 12 (30) 200−500: 19 (47.5) >500: 9 (22.5)	On ART: *N* = 74 (100)	*N* = 131 Male: *N* = 59 (45.0) Female: *N* = 72 (55.0)	18–35 years: *N* = 42 (32.8) 36–50 years: *N* = 64 (50) >50 years: *N* = 22 (17.2)

Pereira et al. [19] (2023)	Brazil	Cross-sectional	*N* = 100 Male = 55 (55) Female = 45 (45)	Mean: 41.03	HIV viral load: undetectable (<40 copies/mL): 100 CD4 count (cells/mm^3^): mean 556.03	On ART: *N* = 100 (100)	*N* = 100 Male = 34 (34) Female = 66 (66)	Mean: 44.81

^1^Country where the study was developed. *Abbreviations*. ART, antiretroviral therapy; AIDS, acquired immunodeficiency syndrome; ARC, AIDS-related complex; cp/mL, copies/mL; HIV, human immunodeficiency virus; NA, not available; PD, periodontal disease; PH, periodontal health; and PDU, parenteral drug users.

**Table 2:** Periodontal profile assessment of the 23 selected studies, classified by chronological order, comparing HIV-positive, and HIV-negative subjects regarding periodontal parameters.

Authors (year)	HIV positive							HIV negative						
	Tobacco use	PPD (mm)	BOP	CAL (mm)	GI	PI	Periodontal status	Tobacco use	PPD (mm)	BOP	CAL (mm)	GI	PI	Periodontal status
Lucht et al. [18] (1991)	NA	Mean HIV-pos: 2.8 ARC: 3.3 AIDS: 3.5	NA	NA	NA	Mean HIV-pos: 28.6% ARC: 26.5% AIDS: 36.1%	NA	NA	Mean: 2.7	NA	NA	NA	Mean: 43.7%	NA

Grbic et al. [27] (1995)	NA	Mean > 4 mm HIV: 24.2 HIV + PDU: 34.0	Mean HIV: 22.7% HIV + PDU: 31.2%	NA	NA	NA	NA	NA	Mean > 4 mm 21.4 PDU: 36.7	Mean 24.9% PDU: 40.8%	NA	NA	NA	NA

Smith et al. [28] (1995)	Smokers: *N* = 14 (48.3%)	NA	Median: 10.70	Sites > 3 mm: 818	NA	Median: 60.3	NA	Smokers: *N* = 15 (55.6%)	NA	Median: 8.30	Sites > 3 mm: 295	NA	Median 60.0	NA

Robinson et al. [26] (1996)	NA	Sites ≥4 mm: *N* = 159 (51.0%)	Sites: *N* = 301 (96.5%)	Sites ≥ 4 mm: *N* = 186 (59.6%)	Mean score: 2 (63.8%)	NA	PD: 159 (51) PH: 153 (49)	NA	Sites ≥ 4 mm: *N* = 83 (31.9%)	Sites: *N* = 240 (92.3%)	Sites ≥ 4 mm: *N* = 74 (28.5%)	Mean score: 2 (46.1%)	NA	PD: 83 (31.9) PH: 177

Ndiaye et al. [25] (1997)	NA	Mean 2.9	NA	Mean 2.9	Mean percentage of sites with score >1: 44%	Mean 41%	Severe PD: 7	Smokers: *N* = 45 (71)	2.7	NA	2.7	Mean percentage of sites with score >1: 33%	Mean 41%	Severe PD: 3

Scheutz et al. [29] (1997)	NA	Number of HIV individuals with two or more sites with >3 mm: 62 (52.1%) Number of AIDS individuals with two or more sites with >3 mm: 38 (52%)	Number of HIV individuals with two or more sites: 68 (57.14%) Number of AIDS individuals with two or more: 41(56.2%)	Number of HIV individuals with two or more sites with >3 mm: 73(61.3%) Number of AIDS individuals with two or more sites with >3 mm: 49 (67.1%)	NA	NA	NA	NA	Individuals with two or more sites with >3 mm: 70 (44.8%)	Individuals with two or more sites: 76 (48.7%)	Individuals with two or more sites with >3 mm: 96 (61.5%)	NA	NA	NA

Robinson et al. [30] (2000)	Smokers: *N* = 12 (63.2%)	NA	NA	Number of individuals with ≥4 mm: 14 (73.7%)	NA	Mean proportion of surfaces: 0.73	NA	Smokers: *N* = 2 (10.5%)	NA	NA	Number of individuals with ≥4 mm: 3 (15.8%)	NA	Mean proportion of surfaces: 0.63	NA

Mulligan et al. [23] (2004)	Smokers: *N* = 347 (61%) Former: *N* = 84 (15%)	NA	NA	NA	NA	Number of individuals with positive PI: 524 (89.7%)	NA	Smokers: *N* = 102 (69%) Former: *N* = 23 (16%)	NA	NA	NA	NA	Number of individuals with positive PI: 139 (92%)	NA

Alves et al. [20] (2006)	Smokers: *N* = 413 (70%)	Mean: 2.1	NA	Mean: 1.6	NA	NA	NA	Smokers: *N* = 110 (82%)	Mean: 2.0	NA	Mean: 1.2	NA	NA	NA

Botero et al. [31] (2007)	NA	Mean 4.5	Mean 56.1%	NA	Mean score: 1.9	NA	PD: 31	NA	Mean PD: 3.6 PH: 2.4	Mean PD: 40.9% PH: 8.9%	NA	Mean score: PD: 1.6 PH: 0.4	NA	PD: 32 PH: 32

Gonçalves et al. [32] (2007)	Smokers with: PD: 23.5% PH: 32.4%	Mean PD: 2.3 PH: 1.8	Mean PD: 38.9% PH: 14.6%	Mean PD: 2.6 PH: 2.0	NA	Mean PD: 41.2% PH: 20.5%	PD: 37 PH: 35	Smokers with: PD: 28.6% PH: 0.0%	Mean PD: 3.3 PH: 2.0	Mean PD: 44.3% PH: 3.7%	Mean: PD: 3.8 PH: 2.1	NA	Mean PD: 62.9% PH: 17.4%	PD: 49 PH: 51

Brito et al. [33] (2008)	NA	Mean: 3.0	NA	Mean: 3.3	Mean score: 1.56	Mean: 1.4	PD: 32 (100%)	NA	Mean: 3.6	NA	Mean: 3.8	Mean score: 1.55	Mean: 1.22	PD: 16 (100%)

Grande et al. [34] (2008)	Smokers: *N* = 15 (30%)	Mean GI: 1.88 PD: 2.63	NA	Mean GI: 2.25 PD: 3.21	NA	Mean: GI: 43.72% PD: 45.33%	PD: 27 GI: 23	Smokers: *N* = 17 (34%)	Mean 2.98	NA	Mean: 3.45	NA	Mean: 42.23%	PD: 50

Nittayananta et al. [35] (2010)	Smokers with: 73 (46.5%)	Number of individuals with ≥4 mm: 127 (82%)	Number of individuals: 146 (94%)	NA	NA	NA	NA	Smokers: *N* = 34 (68%)	Number of Individuals with ≥4 mm: 45 (85%)	Number of individuals: 46 (96%)	NA	NA	NA	NA

Stojkovic et al. [36] (2011)	Smokers: *N* = 15 (60%)	Mean: 2.2	NA	Mean: 2.6	NA	Mean Males: 94.2% Females: 86.7%	NA	Smokers: *N* = 10 (40%)	Mean: 1.4	NA	Mean: 2.1	NA	Mean Males: 45.7% Females: 42.6%	NA

Khamissa et al. [37] (2012)	Smokers: *N* = 3 (10%)	Mean: 3.205	Mean: 47.3%	NA	NA	Mean: 75.6%	PD: 30 (100%)	Smokers: *N* = 5 (16.7%)	Mean: 3.196	Mean: 50.3%	NA	NA	Mean: 75.2%	PD: 30 (100%)

Lourenço et al. [38] (2013)	Smokers PH: 13% PD: 34%	Mean sites with ≥4 mm: PH: N/R PD: 1.4	Mean sites: PH: 9 PD: 32	NA	NA	NA	PD: 39 PH: 32	Smokers PH: 9% PD: 18%	Mean sites with ≥4 mm: PH: N/R PD: 1.3	Mean sites: PH: 10 PD: 35	NA	NA	NA	PH: 22 PD: 16

Ferreira et al. [21] (2015)	Smokers *N* = 20 (71%)	Mean: 2.5	Mean: 48.3%	Mean: 2.9	NA	Mean: 65.8%	PD: 28 (100%)	Smokers: *N* = 3 (30%)	Mean: 2.7	Mean: 54.3%	Mean: 3.2	NA	Mean: 60.3%	PD: 10 (100%)

Ferreira et al. [22] (2016)	NA	Mean: 2.7	Mean: 34.8%	Mean: 3.0	NA	Mean: 58%	PD: 11 (100%)	NA	Mean: 3.7	Mean: 63.4%	Mean: 4.0	NA	Mean: 75.8%	PD: 21 (100%)

Menezes et al. [39] (2018)	Smokers: 0%	Mean: 6.6	NA	Mean: 7.1	NA	NA	PD: 35 (100%)	NA	Mean: 5.9	NA	Mean: 6.4	NA	NA	PD: 35 (100%)

Spolsky et al. [24] (2018)	Smokers: *N* = 86 (61.4%) Former: *N* = 20 (14.3%)	NA	NA	Mean: 2.58	NA	NA	NA	Smokers: *N* = 286 (70.4%) Former: *N* = 32 (7.9%)	NA	NA	Mean: 2.66	NA	NA	NA

Gonçalves et al. [40] (2022)	Smokers: *N* = 32 (45.7%)	≥3 sites with ≥ 5 mm: 37 (50%)	>10%: 57 (77%)	NA	NA	>10%: 58 (78.4%)	PH: 37 (50%) PD: 37 (50%)	Smokers: *N* = 37 (28.9)	≥3 sites with ≥ 5 mm: 72 (55%)	>10%: 74 (56.5%)	NA	NA	>10%: 108 (83.1%)	PH: 59 (45%) PD: 72 (55%)

Pereira et al. [19] (2023)	Smokers: *N* = 84 (84%) Former: *N* = 75 (75%)	NA	Mean: 40.67%	NA	NA	Mean: 41.74%	PH: 46 (46%) PD: 54 (54%)	Smokers: *N* = 12 (12%) Former: *N* = 16 (16%)	NA	Mean: 38.33%	NA	NA	Mean: 45.12%	PH: 73 (73%) PD: 27 (27%)

*Abbreviations*. ART, antiretroviral therapy; AIDS, acquired immunodeficiency syndrome; ARC, AIDS-related complex; cp/mL, copies/mL; GI, gingivitis; HIV, human immunodeficiency virus; NA, not available; PH, periodontal health; PD, periodontal disease; PH, periodontal health; and PDU, parenteral drug users.

## Data Availability

The collected data used to support the findings of this study are included within the article and in Supplementary Materials.

## Supplementary Materials

Supplementary 1 Search strategies used for each database.

Supplementary 2 Excluded records (*N* = 130) and reasons for exclusion.

Supplementary 3 Quality of assessment (QoA) summary for each cross-sectional (A) and cohort studies (B), analyzed according to the Joanna Brigs Institute (JBI) checklist.
