# Supplementary material for: HIV-Infected Individuals Do Not Present Significant Differences regarding Periodontal Status: A Systematic Review and Meta-Analysis
Source: Int J Dent. 2024 Aug 26;2024:5559610. doi: 10.1155/2024/5559610 (PMC11368558; doi:10.1155/2024/5559610)
Supplement: Supplementary 1 — Search strategies used for each database. [file 5559610.f1.docx]

**Supplementary Material 1.** Search strategies used for each database.

| **Database** | **Search strategy: May/2020 - Update July/2023** |
| --- | --- |
| **PubMed** | ((("hiv"[MeSH Terms] OR "hiv"[All Fields]) OR "human immunodeficiency virus"[All Fields] OR ("human"[All Fields] AND "immunodeficiency"[All Fields] AND "virus"[All Fields]) OR "hiv infections"[MeSH Terms] OR "hiv infections"[All Fields] OR ("hiv"[All Fields] AND "infection*"[All Fields]) OR "hiv seropositivity"[MeSH Terms] OR "hiv seropositivity"[All Fields] OR ("hiv"[All Fields] AND "seropositivity"[All Fields]) OR “hiv infected patients”[All Fields] OR (("hiv"[MeSH Terms] OR "hiv"[All Fields]) AND infected[All Fields] AND ("patients"[MeSH Terms] OR "patients"[All Fields])))) AND (“periodontal clinical indices”[All Fields] OR (periodontal[All Fields] AND clinical[All Fields] AND indices[All Fields]) OR “periodontal status” OR (periodontal[All Fields] AND status[All Fields]) OR "periodontal index"[MeSH Terms] OR "periodontal index"[All Fields] OR (“periodontal”[All Fields] AND “indices”[All Fields]) OR “probing pocket depth” [All Fields] OR (probing[All Fields] AND pocket[All Fields] AND depth[All Fields]) OR “bleeding on probing” OR ("bleeding”[All Fields] AND “probing”[All Fields]) OR "plaque index"[All Fields] OR (“plaque”[All Fields] AND “index”[All Fields]) OR "dental plaque"[All Fields] OR (“dental”[All Fields] AND “plaque”[All fields]) OR “gingival index”[All fields] OR (“gingival”[All Fields] AND “index”[All Fields]) OR “clinical attachment level”[All Fields] OR (clinical[All Fields] AND "attachment"[All Fields] AND level[All Fields]) OR “periodontal attachment loss”[All Fields] OR (periodontal[All Fields] AND "attachment"[All Fields] AND loss[All Fields]) OR “periodontal health”[All Fields] OR (periodontal[All Fields] AND ("health"[MeSH Terms] OR "health"[All Fields])) OR "periodontal diseases"[MeSH Terms] OR "periodontal diseases"[All Fields] OR ("periodontal"[All Fields] AND (“disease” [All Fields] OR “diseases” [All Fields])) OR "periodontitis"[MeSH Terms] OR "periodontitis"[All Fields]) |
| **EMBASE** | ''hiv' OR 'hiv'/exp OR hiv OR 'human immunodeficiency virus'/exp OR 'human immunodeficiency virus' OR 'human immunodeficiciency virus' OR (('human' OR 'human'/exp OR human) AND ('immunodeficiency' OR 'immunodeficiency'/exp OR immunodeficiency) AND ('virus' OR 'virus'/exp OR virus)) OR 'hiv infections'/exp OR 'hiv infections' OR (('hiv' OR 'hiv'/exp OR hiv) AND ('infections' OR 'infections'/exp OR infection*)) OR 'hiv seropositivity'/exp OR 'hiv seropositivity' OR (('hiv' OR 'hiv'/exp OR hiv) AND ('seropositivity' OR 'seropositivity'/exp OR seropositivity)) OR ‘hiv infected patient*’ OR (('hiv' OR 'hiv'/exp OR hiv) AND infected AND ('patients'/exp OR 'patients' OR patient*)) AND ‘periodontal clinical indices’ OR (periodontal AND ('clinical' OR 'clinical'/exp OR clinical) AND índices) OR ‘periodontal status’ OR (periodontal AND status) OR 'periodontal index' OR (periodontal AND ('index' OR 'index'/exp OR index)) OR ‘probing pocket depth’ OR (('probing' OR 'probing'/exp OR probing) AND ('pocket' OR 'pocket'/exp OR pocket) AND depth) OR ‘bleeding on probing’ OR (('bleeding' OR 'bleeding'/exp OR bleeding) AND ('probing' OR 'probing'/exp OR probing)) OR 'plaque index'/exp OR 'plaque index' OR 'dental plaque'/exp OR 'dental plaque' OR 'gingival index'/exp OR 'gingival index' OR ‘clinical attachment level’ OR (('clinical' OR 'clinical'/exp OR clinical) AND ('attachment' OR 'attachment'/exp OR attachment) AND level) OR ‘periodontal attachment loss’ OR (periodontal AND ('attachment' OR 'attachment'/exp OR attachment) AND loss) OR ‘periodontal health’ OR (periodontal AND ('health' OR 'health'/exp OR health)) OR ‘periodontal disease*’ OR (periodontal AND ('diseases' OR 'diseases'/exp OR disease*)) OR 'periodontitis' OR 'periodontitis'/exp OR periodontitis |
| **Web of Science** | (hiv OR "human immunodeficiency virus" OR (human AND immunodeficiency AND virus) OR "hiv infections" OR (hiv AND infection*) OR "hiv seropositivity" OR (hiv AND seropositivity) OR “hiv infected patient*” (hiv AND infected AND patient*)) AND (“periodontal clinical indices” OR (periodontal AND clinical AND indices) OR “periodontal status” OR (periodontal AND status) OR "periodontal index" OR “probing pocket depth” OR (probing AND pocket AND depth) OR “bleeding on probing” OR (bleeding AND probing) OR "plaque index" OR "dental plaque" OR “gingival index” OR (gingival AND index) OR “clinical attachment level” OR (clinical AND attachment AND level) OR “periodontal attachment loss” OR (periodontal AND attachment AND loss) OR “periodontal health” OR (periodontal AND health) OR "periodontal diseases" OR (periodontal AND disease*) OR periodontitis) |
| **SCOPUS** | (TITLE-ABS-KEY((hiv OR "human immunodeficiency virus"  OR  ( human  AND  immunodeficiency AND virus) OR "hiv infections" OR (hiv AND infection*) OR "hiv seropositivity" OR (hiv AND seropositivity) OR “hiv infected patient*” OR (hiv AND infected AND patient*)))) AND (TITLE-ABS-KEY(((periodontal AND clinical AND indices) OR “periodontal status” OR (periodontal AND status) OR "periodontal index" OR “probing pocket depth” OR (probing AND pocket AND depth) OR “bleeding on probing” OR (bleeding AND probing) OR "plaque index" OR "dental plaque" OR “gingival index” OR (gingival AND index) OR “clinical attachment level” OR (clinical AND attachment AND level) OR “periodontal attachment loss” OR (periodontal AND attachment AND loss) OR “periodontal health” OR (periodontal AND health) OR "periodontal diseases" OR (periodontal AND disease*) OR periodontitis))) |
| **DOSS (EBSCOhost)** | (hiv OR "human immunodeficiency virus" OR (human AND immunodeficiency AND virus) OR "hiv infections" OR (hiv AND infection*) OR "hiv seropositivity" OR (hiv AND seropositivity) OR “hiv infected patient*” OR (hiv AND infected AND patient*)) AND (“periodontal clinical indices” OR (periodontal AND clinical AND indices) OR “periodontal status” OR (periodontal AND status) OR "periodontal index" OR “probing pocket depth” OR (probing AND pocket AND depth) OR “bleeding on probing” OR (bleeding AND probing) OR "plaque index" OR "dental plaque" OR “gingival index” OR (gingival AND index) OR “clinical attachment level” OR (clinical AND attachment AND level) OR “periodontal attachment loss” OR (periodontal AND attachment AND loss) OR “periodontal health” OR (periodontal AND health) OR "periodontal diseases" OR (periodontal AND disease*) OR periodontitis) |
| **LILACS** | (hiv OR vih) OR ("human immunodeficiency virus" OR "vírus da imunodeficiência humana" OR "vírus de imunodeficiência humana") OR ("hiv infections" OR "infecções por hiv" OR "infecção por hiv" OR "infecciones por vih") OR ("hiv seropositivity" OR "soropositividade para hiv" OR "seropositividad para vih") OR ("hiv infected patients" OR "pacientes infectados por hiv" OR "pacientes infectados por el vih") AND ("periodontal index" OR "índice periodontal") OR ("probing pocket depth" OR "profundidade de sondagem" OR “profundidad de sondaje”) OR ("periodontal attachment loss" OR "perda da inserção periodontal" OR "pérdida de la inserción periodontal") OR ("clinical attachment level" OR "nível clínico de inserção" OR "nivel de inserción clínica") OR ("dental plaque index" OR "índice de placa dentária" OR "índice de placa dental") OR (“periodontal status” OR “estado periodontal” OR “condição periodontal”) OR ("periodontal diseases" OR "doenças periodontais" OR "enfermedades periodontales") OR (periodontitis OR periodontite) |
| **Google Scholar** | allintitle: hiv OR "human immunodeficiency virus": "periodontal status" OR "periodontal health" OR “periodontal clinical indices” OR “periodontal index” |
