# Supplementary material for: HIV-Infected Individuals Do Not Present Significant Differences regarding Periodontal Status: A Systematic Review and Meta-Analysis
Source: Int J Dent. 2024 Aug 26;2024:5559610. doi: 10.1155/2024/5559610 (PMC11368558; doi:10.1155/2024/5559610)
Supplement: Supplementary 2 — Excluded records (N = 130) and reasons for exclusion. [file 5559610.f2.docx]

**Supplementary Material 2.** Excluded records N=130 and reasons for exclusion

| **1.Inadequate control group** | **2.Inadequate report of periodontal parameters** | **3. Inadequate type of study or methodology** |
| --- | --- | --- |
| Alpagot et al., 2004 | Baqui et al., 2000 | Barr et al., 1992 |
| Alpagot et al., 2006 | Barr et al., 1996 | Berniyanti et al., 2019 |
| Alpagot et al., 2008 | Choromańska & Waszkiel, 2006 | Filho & Giovani, 2009 |
| Asif et al., 2012 | Cross & Smith, 1995 | Dubois et al., 2020 |
| Fuentes Ayala et al., 2011 | Doshi et al., 2008 | Escalona et al., 2011 |
| Azatyan et al., 2021 | Engeland et al., 2008 | Escalona et al., 2016 |
| Baqui et al.^6^ | Falasca et al., 2008 | Ferreira et al., 2018 |
| Barbi et al., 2021 | Griffen et al., 2019 | Gladys et al., 2018 |
| Diniz Barreto et al., 2016 | Guarnelli et al., 1999 | Gonçalves et al., 2006 |
| Brown et al., 2002 | Kumar et al., 2014 | Gonçalves et al., 2009 |
| Cao et al., 2022 | Lamster et al., 1994 | De Souza Gonçalves et al., 2009 |
| Chaudhary et al., 2020 | Melnick et al., 1989 | Gornitsky et al., 1991 |
| Coates et al., 1996 | Noguera-Julian et al., 2017 | Grbic et al., 1997 |
| Doshi et al., 2008 | Nouaman et al., 2015 | Groenewegen et al., 2019 |
| Dragonas et al., 2018 | Peppes et al., 2013 | Güarinos et al., 1996 |
| Drinkard et al., 1991 | Persson et al., 1998 | Hofer, 1996 |
| Fokam et al., 2020 | Ranganathan et al., 2012 | Hofer et al., 2002 |
| Fricke et al., 2012 | Scully et al., 1999 | Holmström et al., 1990 |
| Friedman et al., 1991 | Umeizudike et al., 2014 | Janorkar et al., 2022 |
| Gaetti-Jardim et al., 2008 | Vernon et al., 2019 | Jordan et al., 2006 |
| Gliosca et al., 2019 | Yeung et al., 2002 | Klimiuk et al., 2006 |
| Gonçalves et al., 2004 |  | Lourenco et al., 2014 |
| Grande et al., 2011 |  | Lourenço et al., 2017 |
| Groenewegen et al., 2022 |  | Mellanen et al., 1996 |
| Guerra et al., 2013 |  | Menezes et al., 2018 |
| Guimarães et al., 2012 |  | Mulligan et al., 2008 |
| Hegde et al., 2016 |  | Niazi et al., 2020 |
| Ilenko-Lobach et al., 2022 |  | Nobre et al., 2019 |
| Induchoodan, 2008 |  | Nobre et al., 2020 |
| Jácome-Santos et al., 2020 |  | Noro Filho et al., 2012 |
| Jana et al., 2022 |  | Mello Silva et al., 2012 |
| John et al., 2012 |  | Williams‐Wiles & Vieira, 2019 |
| John et al., 2013 |  |  |
| Jordan et al., 2018 |  |  |
| Kheur et al., 2021 |  |  |
| Kiran, Karnam, & Niharika, 2022 |  |  |
| Kiran, Karnam, Ramya, et al., 2022 |  |  |
| Kosandal et al., 2013 |  |  |
| Lam et al., 2022 |  |  |
| Lemos et al., 2010 |  |  |
| Lomeli-Martinez et al., 2022 |  |  |
| Lustosa de Souza et al., 2023 |  |  |
| Maloth et al., 2020 |  |  |
| Martínez-Canut et al., 1996 |  |  |
| Masouredis et al., 1992 |  |  |
| Matičić et al., 2000 |  |  |
| McKaig et al., 1998 |  |  |
| McKaig et al., 2000 |  |  |
| Mehlotra et al., 2016 |  |  |
| Muralidharan, Acharya, Margabandhu, et al., 2018 |  |  |
| Muralidharan, Acharya, Sevekari, et al., 2018 |  |  |
| Ríos et al., 2002 |  |  |
| Noro Filho et al., 2013 |  |  |
| Odden et al., 1994 |  |  |
| Pavan et al., 2014 |  |  |
| Price et al., 1999 |  |  |
| Gontán Quintana et al., 2013 |  |  |
| Rai et al., 2022 |  |  |
| Ranganathan et al., 2007 |  |  |
| Riley et al., 1992 |  |  |
| Rozra et al., 2012 |  |  |
| Sampath et al., 2022 |  |  |
| Santo et al., 2010 |  |  |
| Schuettfort et al., 2018 |  |  |
| Sehdev et al., 2019 |  |  |
| Shaghaghian et al., 2021 |  |  |
| Shugars et al., 2000 |  |  |
| Tomar et al., 1995 |  |  |
| Trentin et al., 2007 |  |  |
| Tukutuku et al., 1990 |  |  |
| Teanpaisan et al., 2001 |  |  |
| Umeizudike et al., 2014 |  |  |
| Vastardis et al., 2003 |  |  |
| Vernon et al., 2013 |  |  |
| Vernon et al., 2019 |  |  |
| Wulandari et al., 2020 |  |  |
| Yeung et al., 1993 |  |  |

**REFERENCES**

Alpagot, T., Duzgunes, N., Wolff, L. F., & Lee, A. 2004. Risk factors for periodontitis in HIV+ patients. *Journal of Periodontal Research*, *39*3, 149–157. https://doi.org/10.1111/j.1600-0765.2004.00718.x

Alpagot, T., Konopka, K., Bhattacharyya, M., Gebremedhin, S., & Düzgüneş, N. 2008. The Association Between Gingival Crevicular Fluid TGF-β1 Levels and Periodontal Status in HIV-1 + Patients. *Journal of Periodontology*. https://doi.org/10.1902/jop.2008.070312

Alpagot, T., Suzara, V., & Bhattacharyya, M. 2006. The associations between gingival crevice fluid matrix metalloproteinase-9, tissue inhibitor of metalloproteinase-1 and periodontitis in human immunodeficiency virus-positive patients. *Journal of Periodontal Research*, *41*6. https://doi.org/10.1111/j.1600-0765.2006.00887.x

Asif, K., Kothiwale, S., Neelima, K., & Patil, R. 2012. Periodontal status in HIV-positive individuals and its possible correlation with CD4+T cell count. *Chronicles of Young Scientists*, *3*2. https://doi.org/10.4103/2229-5186.98689

Azatyan, V., Yessayan, L., Aznauryan, A., & Porkeshyan, K. 2021. [ORAL HEALTH STATUS IN HIV-POSITIVE PATIENTS]. *Georgian Medical News*, *314*, 56–63.

Baqui, A. A. M. A., Meiller, T. F., Jabra-Risk, M. A., Zhang, M., Keller, J. I., & Falker Jr, W. A. 2000. Enhanced interleukin 1α, interleukin 6 and tumor necrosis factor a in gingival crevicular fluid from periodontal pockets of patients infected with human immunodeficiency virus 1. *Oral Microbiology & Immunology*, *15*2, 67–73.

Barbi, W., Shalini, K., Kumari, A., Raaj, V., Gupta, H., Gauniyal, P., & Rangari, P. 2021. Assessment of oral health and prevalence of oral conditions in human immunodeficiency virus-infected subjects visiting antiretroviral therapy centers. *Journal of Pharmacy And Bioallied Sciences*, *13*6, 1470. https://doi.org/10.4103/jpbs.jpbs_256_21

Barr, C. E., Qureshi, M. N., Qiu, Z., Kaim, J., & Zhang, W. 1996. Oral HIV-I recovery in the presence of periodontal disease. *Oral Diseases*, *2*3. https://doi.org/10.1111/j.1601-0825.1996.tb00224.x

Barr, C., Lopez, M. R., & Rua-Dobles, A. 1992. Periodontal changes by HIV serostatus in a cohort of homosexual and bisexual men. *Journal of Clinical Periodontology*, *19*10, 794–801. https://doi.org/10.1111/j.1600-051X.1992.tb02173.x

Berniyanti, T., Setijanto, R. D., Hariani, N., & Romadhoni, S. F. 2019. Relationship between HIV/AIDS and periodontal health status among population at high risk of HIV/AIDS. In *Acta Medica Philippina* Vol. 53, Issue 5. https://doi.org/10.47895/amp.v53i5.97

Brown, J. B., Rosenstein, D., Mullooly, J., O’Keeffe Rosetti, M., Robinson, S., & Chiodo, G. 2002. Impact of intensified dental care on outcomes in human immunodeficiency virus infection. *AIDS Patient Care and STDs*, *16*10. https://doi.org/10.1089/10872910260351258

Cao, P., Zhang, Y., Dong, G., Wu, H., Yang, Y., & Liu, Y. 2022. Clinical Oral Condition Analysis and the Influence of Highly Active Antiretroviral Therapy on Human Salivary Microbial Community Diversity in HIV-Infected/AIDS Patients. *Frontiers in Cellular and Infection Microbiology*, *12*. https://doi.org/10.3389/fcimb.2022.937039

Chaudhary, P., Manral, K., Gupta, R., Bengani, A. S., Chauhan, B., & Arora, D. 2020. Oral health status and treatment needs among HIV/AIDS patients attending antiretroviral therapy center in Western India: A cross-sectional study. *Journal of Family Medicine and Primary Care*, *9*7, 3722. https://doi.org/10.4103/jfmpc.jfmpc_411_20

Choromańska, M., & Waszkiel, D. 2006. Periodontal status and treatment needs in HIV-infected patients. *Advances in Medical Sciences*, *51 Suppl 1*.

Coates, E., Slade, G. D., Goss, A. N., & Gorkic, E. 1996. Oral conditions and their social impact among HIV dental patients. *Australian Dental Journal*, *41*1. https://doi.org/10.1111/j.1834-7819.1996.tb05652.x

Cross, D. L., & Smith, G. L. F. 1995. Comparison of periodontal disease in HIV seropositive subjects and controls ll.. Microbiology, immunology and predictors of disease progression. *Journal of Clinical Periodontology*, *22*7, 569–577. https://doi.org/10.1111/j.1600-051X.1995.tb00806.x

de Mello Silva, A., dos Santos, C. C., & Giovani, E. M. 2012. Diagnosis of oral manifestations in HIV/AIDS patients who used HAART and developed diabetes mellitus. *Brazilian Journal of Oral Sciences*, *11*3.

de Menezes, S. A. F., Menezes, T. O. de A., Rodrigues, T. M. de S., Nogueira, B. M. L., & Fonseca, R. R. de S. 2018. Analysis of IL-10 in HIV-1 patients with chronic periodontitis in northern Brazil. *Brazilian Journal of Oral Sciences*, *16*. https://doi.org/10.20396/BJOS.V16I0.8651054

De S. Gonçalves, L., Ferreira, S. M. S., Souza, C. O., & Colombo, A. P. V. 2006. IL-1 gene polymorphism and periodontal status of HIV Brazilians on highly active antiretroviral therapy. *AIDS*, *20*13. https://doi.org/10.1097/01.aids.0000242826.97495.7c

De Souza Gonçalves, L., Souto, R., & Colombo, A. P. V. 2009. Detection of helicobacter pylori, enterococcus faecalis, and pseudomonas aeruginosa in the subgingival biofilm of hiv-infected subjects undergoing HAART with chronic periodontitis. *European Journal of Clinical Microbiology and Infectious Diseases*, *28*11. https://doi.org/10.1007/s10096-009-0786-5

Diniz Barreto, L. P., Melo dos Santos, M., Gomes, B. da S., Lamas, C. da C., Silva, D. G. da, Silva-Boghossian, C. M., Soares, L. G., & Vieira Falabella, M. E. 2016. Periodontal Conditions in Human Immunodeficiency Virus–Positive Patients Under Highly Active Antiretroviral Therapy From a Metropolitan Area of Rio De Janeiro. *Journal of Periodontology*, *87*4, 338–345. https://doi.org/10.1902/jop.2015.150345

Doshi, D., Ramapuram, J. T., Anup, N., & Sharma, G. 2008. Correlation of CD4 cell count with gingival bleeding index in HIV positive individuals. *Medicina Oral, Patologia Oral y Cirugia Bucal*, *13*6.

Dragonas, P., Kaste, L. M., Nunn, M., Gajendrareddy, P. K., Weber, K. M., Cohen, M., Adeyemi, O. M., French, A. L., & Sroussi, H. Y. 2018. Vitamin D deficiency and periodontal clinical attachment loss in HIV-seropositive women: A secondary analysis conducted in the Women’s Interagency HIV Study WIHS. *Oral Surgery, Oral Medicine, Oral Pathology and Oral Radiology*, *125*6. https://doi.org/10.1016/j.oooo.2018.02.006

Drinkard, C. R., Decher, L., Little, J. W., Rhame, F. S., Balfour, H. H., Rhodus, N. L., Merry, J. W., Walker, P. O., Miller, C. E., Volberding, P. A., & Melnick, S. L. 1991. Periodontal status of individuals in early stages of human immunodeficiency virus infection. *Community Dentistry and Oral Epidemiology*, *19*5. https://doi.org/10.1111/j.1600-0528.1991.tb00168.x

Dubois, V. A., González, M. I., Martínez, M. E., Fedelli, L., Lamas, S., D Eramo, L. R., Squassi, A. F., Sánchez, G. A., Salgado, P., Gliosca, L. A., & Molgatini, S. L. 2020. Enzyme production by Candida albicans and Candida dubliniensis in periodontal HIV-positive patients receiving and not receiving antiretroviral therapy. *Acta Odontologica Latinoamericana : AOL*, *33*2, 104–111.

Engeland, C. G., Jang, P., Alves, M., Marucha, P. T., & Califano, J. 2008. HIV infection and tooth loss. *Oral Surgery, Oral Medicine, Oral Pathology, Oral Radiology and Endodontology*, *105*3. https://doi.org/10.1016/j.tripleo.2007.10.012

Escalona, L., Correnti, M., Veitia, D., & Perrone, M. 2011. Detection of human papillomavirus in gingival fluid of Venezuelan HIV patients with periodontal disease. *Investigacion Clinica*, *52*3.

Escalona, L., Veitía, D., & Correnti, M. 2016. Detection of ebv, CMV and HSV-1 in subgingival samples of HIV positive and negative patients with chronic periodontitis. *Journal of Oral Research*, *5*4. https://doi.org/10.17126/joralres.2016.036

Falasca, K., Vecchiet, F., Ucciferri, C., Vignale, F., Conti, P., Pizzigallo, A., Piatelli, A., & Vecchiet, J. 2008. Periodontitis and cytokine patterns in HIV positive patients. *European Journal of Medical Research*, *13*4.

Ferreira, M. V. M., Cavalcanti, É. F. F., De Pm Rubini, N., Ferreira, D. C., Gonçalves, L. S., & Colombo, A. P. V. 2018. Oral status and periodontal microbiota of HIV-infected youth infected by vertical transmission. *Future Virology*, *13*4. https://doi.org/10.2217/fvl-2018-0025

Filho, J. C. C., & Giovani, É. M. 2009. Xerostomy, dental caries and periodontal disease in HIV+ patients. *Brazilian Journal of Infectious Diseases*, *13*1. https://doi.org/10.1590/s1413-86702009000100005

Fokam, J., Geh, B. K. N., Sosso, S. M., Takou, D., Ngufack, E. S., Nka, A. D., Bissek, A.-C. Z.-K., Eko, D. M., & Ndjolo, A. 2020. Determinants of periodontitis according to the immunological and virological profiles of HIV-infected patients in Yaoundé, Cameroon. *BMC Oral Health*, *20*1, 359. https://doi.org/10.1186/s12903-020-01353-7

Fricke, U., Geurtsen, W., Staufenbiel, I., & Rahman, A. 2012. Periodontal status of HIV-infected patients undergoing antiretroviral therapy compared to HIV-therapy naive patients: A case control study. *European Journal of Medical Research*, *17*1. https://doi.org/10.1186/2047-783X-17-2

Friedman, R. B., Gunsolley, J., Gentry, A., Dinius, A., Kaplowitz, L., & Settle, J. 1991. Periodontal Status of HIV-Seropositive and AIDS Patients. *Journal of Periodontology*, *62*10. https://doi.org/10.1902/jop.1991.62.10.623

Fuentes Ayala, E., Hernández Rodríguez, Y., Pérez Hernández, L. Y., & Hernández González, P. L. 2011. Salud periodontal en personas viviendo con VIH/sida. Pinar del Río, 2008 TT - Periodontal health in people living with HIV/AIDS in Pinar del Rio, 2008. *Revista de Ciencias Médicas de Pinar Del Río*, *15*4.

Gaetti-Jardim, E., Nakano, V., Wahasugui, T. C., Cabral, F. C., Gamba, R., & Avila-Campos, M. J. 2008. Occurrence of yeasts, enterococci and other enteric bacteria in subgingival biofilm of HIV-positive patients with chronic gingivitis and necrotizing periodontitis. *Brazilian Journal of Microbiology*, *39*2. https://doi.org/10.1590/S1517-83822008000200011

Gladys, A., Kituku, M., James, R. M., & Hudson, A. 2018. Periodontal status of HIV Discordant Couples. *Journal of Dental and Medical Sciences*, *17*10, 36–40.

Gliosca, L. A., D Eramo, L. R., Bozza, F. L., Soken, L., Abusamra, L., Salgado, P. A., Squassi, A. F., & Molgatini, S. L. 2019. Microbiological study of the subgingival biofilm in HIV+/HAART patients at a specialized dental service. *Acta Odontologica Latinoamericana : AOL*, *32*3.

Gonçalves, L. D. S., Ferreira, S. M. S., Souza, C. O., & Colombo, A. P. V. 2009. Influence of IL-1 gene polymorphism on the periodontal microbiota of HIV-infected Brazilian individuals. *Brazilian Oral Research*, *23*4, 452–459. https://doi.org/10.1590/S1806-83242009000400016

Gonçalves, L. de S., Ferreira, S. M., Silva, A., Villoria, G. E., Costinha, L. H., Souto, R., Uzeda, M. de, & Colombo, A. P. 2004. Association of T CD4 lymphocyte levels and subgingival microbiota of chronic periodontitis in HIV-infected Brazilians under HAART. *Oral Surgery, Oral Medicine, Oral Pathology, Oral Radiology, and Endodontology*, *97*2, 196–203. https://doi.org/10.1016/j.tripleo.2003.08.023

Gontán Quintana, N., Soto Ugalde, A., & Otero Salabarría, E. 2013. Enfermedad periodontal inflamatoria crónica en pacientes diagnosticados con virus de inmunodeficiencia humana/sida en Cienfuegos. *MediSur*, *11*4.

Gornitsky, M., Clark, D. C., Siboo, R., Amsel, R., lugovaz, I., Wooley, C., luliani, N., & Chan, E. C. S. 1991. Clinical Documentation and Occurrence of Putative Periodontopathic Bacteria in Human Immunodeficiency Virus-Associated Periodontal Disease. *Journal of Periodontology*, *62*9. https://doi.org/10.1902/jop.1991.62.9.576

Grande, S. R., Imbronito, A. V., Okuda, O. S., Pannuti, C. M., Nunes, F. D., & Lima, L. A. 2011. Relationship Between Herpesviruses and Periodontopathogens in Patients With HIV and Periodontitis. *Journal of Periodontology*, *82*10. https://doi.org/10.1902/jop.2011.100723

Grbic, J. T., Lamster, I. B., & Mitchell-Lewis, D. 1997. Inflammatory and Immune Mediators in Crevicular Fluid From HIV-Infected Injecting Drug Users. *Journal of Periodontology*, *68*3. https://doi.org/10.1902/jop.1997.68.3.249

Griffen, A. L., Thompson, Z. A., Beall, C. J., Lilly, E. A., Granada, C., Treas, K. D., DuBois, K. R., Hashmi, S. B., Mukherjee, C., Gilliland, A. E., Vazquez, J. A., Hagensee, M. E., Leys, E. J., & Fidel, P. L. 2019. Significant effect of HIV/HAART on oral microbiota using multivariate analysis. *Scientific Reports*, *9*1, 19946. https://doi.org/10.1038/s41598-019-55703-9

Groenewegen, H., Bierman, W. F. W., Delli, K., Dijkstra, P. U., Nesse, W., Vissink, A., & Spijkervet, F. K. L. 2019. Severe periodontitis is more common in HIV- infected patients. *Journal of Infection*, *78*3, 171–177. https://doi.org/10.1016/j.jinf.2018.11.008

Groenewegen, H., Delli, K., Vissink, A., Spijkervet, F. K. L., & Bierman, W. F. W. 2022. Immune markers and microbial factors are related with periodontitis severity in people with HIV. *Clinical Oral Investigations*, *27*3, 1255–1263. https://doi.org/10.1007/s00784-022-04758-6

Güarinos, J., Bagán, J. V., & Martínez-Canut, P. 1996. Dental health in patients infected by human immunodeficiency virus HIV. A study of 94 cases. *Bulletin Du Groupement International Pour La Recherche Scientifique En Stomatologie & Odontologie*, *39*3–4.

Guarnelli, M. E., Trombelli, L., & Calura, G. 1999. Radiographic evaluation of alveolar bone height in HIV-positive patients. *Minerva Stomatologica*, *48*6.

Guerra, M. E., Guerra, A., Zamora, M., Castillo, T., Carrasco, W., & Osorio, A. Y. 2013. Enfermedad periodontal en mujeres vih/sida TT  - Periodontal disease in women HIV / AIDS. *Acta Odontol Venez*, *51*1.

Guimarães, G., Franco, G. C. N., Cortelli, J. R., Cogo, K., Costa, F. O., Aquino, D. R., Lustosa, A., & Cortelli, S. C. 2012. Portadores do HIV apresentam pior condição clínica periodontal e maior prevalência de Porphyromonas gingivalis e Tannerella forsythia. *Rev. Odontol. UNESP Online*, *41*1.

Hegde, V., Shetty, P., Alva, S., & Chengappa, Sk. 2016. Assessment of dental caries experience, periodontal status, and oral mucosal lesions among human immunodeficiency virus seropositives with and without antiretroviral therapy: A cross-sectional study. *Journal of Indian Association of Public Health Dentistry*, *14*1. https://doi.org/10.4103/2319-5932.178717

Hofer, D. 1996. The effect of a single mechanical treatment on the subgingival microflora in patients with HIV-associated gingivitis. *Journal of Clinical Periodontology*, *23*3 PART I. https://doi.org/10.1111/j.1600-051x.1996.tb02074.x

Hofer, D., Hämmerle, C. H. F., Grassi, M., & Lang, N. P. 2002. Long-term results of supportive periodontal therapy SPT in HIV-seropositive and HIV-seronegative patients. *Journal of Clinical Periodontology*, *29*7. https://doi.org/10.1034/j.1600-051X.2002.290707.x

Holmström, P., Syrjänen, S., Laine, P., Valle, S. ‐L, & Suni, J. 1990. HIV antibodies in whole saliva detected by ELISA and western blot assays. *Journal of Medical Virology*, *30*4. https://doi.org/10.1002/jmv.1890300403

Ilenko-Lobach, N., Petrushanko, T., Ilenko, N., & Bojchenko, O. 2022. CLINICAL AND HAEMATOLOGICAL CHANGES AMONG HIV PATIENTS. *Georgian Medical News*, *322*, 126–130.

Induchoodan, A. 2008. Periodontal status in stages of HIV disease - a short-term clinical study. *Periodontal Practice Today*, *5*4.

Jácome-Santos, H., de Almeida Amanajás, T., Gomes, S. T. M., Machado, L. F. A., Neto, A. R. L. P., de Melo Alves Junior, S., de Jesus Viana Pinheiro, J., Alves, B. P., & Amoras-Alves, A. C. B. 2020. Epstein-Barr virus EBV in periodontal sites of human immunodeficiency virus HIV-positive individuals in North Brazil: A cross-sectional study. *Quintessence International*, *51*1. https://doi.org/10.3290/j.qi.a43616

Jana, P., Sahu, S., Sivaranjini, K., Hamide, A., & Roy, G. 2022. Prevalence of oral lesions and its associated risk factors among PLHIV availing anti-retroviral therapy from a selected tertiary care hospital, Puducherry - A cross sectional analytical study. *Indian Journal of Community Medicine*, *47*2, 235. https://doi.org/10.4103/ijcm.ijcm_850_21

Janorkar, D. A., Long, D. M., Weber, K. M., Sharma, A., Lin, G., D’Souza, G., Edmonds, A., Kassaye, S., Lahiri, C. D., & Konkle‐Parker, D. 2022. Association between BMI and periodontitis in women living with or at risk for HIV. *Special Care in Dentistry*, *42*5, 486–493. https://doi.org/10.1111/scd.12711

John, C. N., Stephen, L. X., & Joyce Africa, C. W. 2013. Is human immunodeficiency virus HIV stage an independent risk factor for altering the periodontal status of HIV-positive patients? A South African study. *BMC Oral Health*, *13*1. https://doi.org/10.1186/1472-6831-13-69

John, C. N., Xavier Graham Stephen, L., & Wilma Joyce Africa, C. 2012. BANA-positive plaque samples are associated with oral hygiene practices and not CD4+ T cell counts in HIV-positive patients. *International Journal of Dentistry*. https://doi.org/10.1155/2012/157641

Jordan, A. R., Gängler, P., & Jöhren, H. P. 2006. Clinical treatment outcomes of periodontal therapy in HIV-seropositive patients undergoing highly active antiretroviral therapy. *European Journal of Medical Research*, *11*6.

Jordan, R. A., Lucaciu, A., Schaper, K., Jöhren, H.-P., & Zimmer, S. 2018. Effectiveness of Systematic Periodontal Treatment in Male HIV-Infected Patients after 9 Years: A Case Series. *Advances in Medicine*, *2018*. https://doi.org/10.1155/2018/4135607

Kheur, S., Kulkarni, M., Mahajan, P. G., Kheur, M., Raj, A. T., Patil, S., & Awan, K. H. 2021. Comparing the sub-gingival levels of Cytomegalovirus, Epstein-Barr virus, Porphyromonas gingivalis in human immunodeficiency virus-1 seropositive patients with and without antiretroviral therapy. *Disease-a-Month*, *67*9, 101166. https://doi.org/10.1016/j.disamonth.2021.101166

Kiran, S., Karnam, Y., & Niharika, B. 2022. Epidemiological evaluation of maternal periodontal status in human immunodeficiency virus seropositive pregnant women in India. *Journal of Indian Association of Public Health Dentistry*, *20*2, 183. https://doi.org/10.4103/jiaphd.jiaphd_97_21

Kiran, S., Karnam, Y., Ramya, Y., Niharika, B., Rani, P., & Sarma, S. 2022. Association between periodontitis and preterm low birth weight in human immunodeficiency virus seropositive pregnant women in India: A cohort study. *Journal of Family Medicine and Primary Care*, *11*12, 7830. https://doi.org/10.4103/jfmpc.jfmpc_1203_22

Klimiuk, A., Waszkiel, D., Choromańska, M., Jankowska, A., & Zelazowska-Rutkowska, B. 2006. The saliva immunology mechanisms and periodontal status in HIV infected subjects. *Advances in Medical Sciences*, *51 Suppl 1*.

Kosandal, K., Totad, S., & Mujawar, S. 2013. Assessment of Oral Diseases and Impact of Antiretroviral Therapy in HIV Population Residing in Southern India. *Indian Journal of Stomatology*, *4*1, 20.

Kumar, S., Mishra, P., Warhekar, S., Airen, B., Jain, D., & Godha, S. 2014. Oral health status and oromucosal lesions in patients living with HIV/AIDS in India: A comparative study. *AIDS Research and Treatment*, *2014*. https://doi.org/10.1155/2014/480247

Lam, P. P. Y., Zhou, N., Wong, H. M., & Yiu, C. K. Y. 2022. Oral Health Status of Children and Adolescents Living with HIV Undergoing Antiretroviral Therapy: A Systematic Review and Meta-Analysis. *International Journal of Environmental Research and Public Health*, *19*19, 12864. https://doi.org/10.3390/ijerph191912864

Lamster, I. B., Begg, M. D., Mitchell-Lewis, D., Fine, J. B., Grbic, J. T., Todak, G. G., El-Sadr, W., Gorman, J. M., Zambon, J. J., & Phelan, J. A. 1994. Oral manifestations of HIV infection in homosexual men and intravenous drug users. Study design and relationship of epidemiologic clinical, and immunologic parameters to oral lesions. *Oral Surgery, Oral Medicine, Oral Pathology*, *78*2. https://doi.org/10.1016/0030-42209490140-6

Lemos, S. S. S., Oliveira, F. A., & Vencio, E. F. 2010. Periodontal disease and oral hygiene benefits in HIV seropositive and AIDS patients. *Medicina Oral, Patologia Oral y Cirugia Bucal*, *15*2. https://doi.org/10.4317/medoral.15.e417

Lomeli-Martinez, S. M., González-Hernández, L. A., Villanueva, J. F. A., Valentín-Goméz, E., Ratkovich-González, S., Alvarez-Zavala, M., Sánchez-Reyes, K., Cabrera-Silva, R. I., & Varela-Hernández, J. J. 2022. In vitro Azole antifungals susceptibility of Candida spp. isolates from HIV-infected patients with periodontitis. *Journal of Medical Mycology*, *32*3, 101294. https://doi.org/10.1016/j.mycmed.2022.101294

Lourenço, A. G., Ribeiro, A. E. R. A., Nakao, C., Motta, A. C. F., Antonio, L. G. L., Machado, A. A., & Komesu, M. C. 2017. Oral Candida spp carriage and periodontal diseases in HIV-infected patients in Ribeirão Preto, Brazil. *Revista Do Instituto de Medicina Tropical de Sao Paulo*, *59*. https://doi.org/10.1590/s1678-9946201759029

Lourenco, A., Ribeiro, A., Nakao, C., Motta, A., Machado, A., & Komesu, M. 2014. Influence of Antiretroviral Therapy and Periodontal Disease on Human Salivary Beta-Defensin 2 in Patients Infected with HIV. *Current HIV Research*, *12*1. https://doi.org/10.2174/1570162x12666140407125120

Lustosa de Souza, B. K., Faé, D. S., Lemos, C. A. A., Verner, F. S., Machado, R. A., Ortega, R. M., & de Aquino, S. N. 2023. Associated oral manifestations with HIV southeastern Brazilian patients on antiretroviral therapy. *Brazilian Journal of Otorhinolaryngology*, *89*3, 425–431. https://doi.org/10.1016/j.bjorl.2023.01.001

Maloth, S., Shrinivas, T., Pramod Krishna, B., & Nagarathna, P. 2020. Prevalence of oromucosal lesions in HIV positive patients receiving haart-A prospective clinical study. *Journal of Family Medicine and Primary Care*, *9*9, 4821. https://doi.org/10.4103/jfmpc.jfmpc_881_20

Martínez-Canut, P., Guarinos, J., & Bagán, J. V. 1996. Periodontal Disease in HIV Seropositive Patients and Its Relation to Lymphocyte Subsets. *Journal of Periodontology*, *67*1. https://doi.org/10.1902/jop.1996.67.1.33

Masouredis, C. M., Katz, M. H., Greenspan, D., Herrera, C., Hollander, H., Greenspan, J. S., & Winkler, J. R. 1992. Prevalence of HIV-Associated Periodontitis and Gingivitis in HIV-Infected Patients Attending an AIDS Clinic. *JAIDS Journal of Acquired Immune Deficiency Syndromes*, *5*5. https://journals.lww.com/jaids/fulltext/1992/05000/prevalence_of_hiv_associated_periodontitis_and.8.aspx

Matičić, M., Poljak, M., Kramar, B., Tomažić, J., Vidmar, L., Zakotnik, B., & Skalerič, U. 2000. Proviral HIV-1 DNA in gingival crevicular fluid of HIV-1-infected patients in various stages of HIV disease. *Journal of Dental Research*, *79*7. https://doi.org/10.1177/00220345000790071101

McKaig, R. G., Patton, L. L., Thomas, J. C., Strauss, R. P., Slade, G. D., & Beck, J. D. 2000. Factors associated with periodontitis in an HIV-infected Southeast USA study. *Oral Diseases*, *6*3. https://doi.org/10.1111/j.1601-0825.2000.tb00327.x

McKaig, R. G., Thomas, J. C., Patton, L. L., Strauss, R. P., Slade, G. D., & Beck, J. D. 1998. Prevalence of HIV-associated periodontitis and chronic periodontitis in a southeastern US study group. *Journal of Public Health Dentistry*, *58*4. https://doi.org/10.1111/j.1752-7325.1998.tb03012.x

Mehlotra, R. K., Hall, N. B., Willie, B., Stein, C. M., Weinberg, A., Zimmerman, P. A., & Vernon, L. T. 2016. Associations of toll-like receptor and β-defensin polymorphisms with measures of periodontal disease PD in HIV+ North American adults: An exploratory study. *PLoS ONE*, *11*10. https://doi.org/10.1371/journal.pone.0164075

Mellanen, L., Ingman, T., Lähdevirta, J., Lauhio, A., Ainamo, A., Konttinen, Y. T., Sukura, A., Salo, T., & Sorsa, T. 1996. Matrix metalloproteinases-I, -3 and -8 and myeloperoxidase in saliva of patients with human immunodeficiency virus infection. *Oral Diseases*, *2*4. https://doi.org/10.1111/j.1601-0825.1996.tb00236.x

Melnick, S. L., Engel, D., Truelove, E., DeRouen, T., Morton, T., Schubert, M., Dunphy, C., & Wood, R. W. 1989. Oral mucosal lesions: Association with the presence of antibodies to the human immunodeficiency virus. *Oral Surgery, Oral Medicine, Oral Pathology*, *68*1. https://doi.org/10.1016/0030-42208990112-6

Mulligan, R., Seirawan, H., Alves, M. E., Navazesh, M., Phelan, J. A., Greenspan, D., Greenspan, J. S., & Mack, W. J. 2008. Oral health-related quality of life among HIV-infected and at-risk women. *Community Dentistry and Oral Epidemiology*, *36*6. https://doi.org/10.1111/j.1600-0528.2008.00443.x

Muralidharan, S., Acharya, A. K., Margabandhu, S., Kalekhan, S., Ahsan, S., & Kulkarni, D. 2018. Prevalence of periodontitis and soft tissue lesions among human immunodeficiency virus-positive patients on antiretroviral therapy in Raichur Taluk, Karnataka, India. *Journal of Contemporary Dental Practice*, *19*1. https://doi.org/10.5005/JP-JOURNALS-10024-2209

Muralidharan, S., Acharya, A., Sevekari, T., Wadwan, S., Joglekar, N. R., & Margabandhu, S. 2018. Prevalence of soft-Tissue lesions among women in sex work in the red light area of Pune, India: A cross-sectional survey. In *Journal of International Society of Preventive and Community Dentistry* Vol. 8, Issue 3. https://doi.org/10.4103/jispcd.JISPCD_46_18

Niazi, F. H., Koppolu, P., Tanvir, S. B., Samran, A., & Alqerban, A. 2020. Clinical efficacy of photodynamic therapy in the treatment of necrotizing ulcerative periodontitis among HIV seropositive patients: A randomized controlled clinical trial. *Photodiagnosis and Photodynamic Therapy*, *29*. https://doi.org/10.1016/j.pdpdt.2019.101608

NOBRE, Á. V. V., DOS SANTOS PÓLVORA, T. L., TIRAPELLI, C., TABA, M., DA CONCEIÇÃO PEREIRA SARAIVA, M., LOURENÇO, A. G., & MOTTA, A. C. F. 2020. PERIODONTAL THERAPY AFFECTS ORAL COLONIZATION OF CANDIDA SPP AND HIV INFECTION STATUS IN PATIENTS WITH PERIODONTITIS. *Oral Surgery, Oral Medicine, Oral Pathology and Oral Radiology*, *129*1. https://doi.org/10.1016/j.oooo.2019.06.761

Nobre, Á. V. V., Pólvora, T. L. S., Silva, L. R. M., Teles, V. de O., Villafuerte, K. V., da Motta, R. J. G., Fortes, J. H. P., Silva, G. A., Ranieri, A. L. P., de Macedo, L. D., Morejon, K. M. L., da Fonseca, B. A. L., Tirapelli, C., Saraiva, M. C. P., Taba, M., Lourenço, A. G., & Motta, A. C. F. 2019. Effects of non-surgical periodontal therapy on clinical and immunological profile and oral colonization of Candida spp in HIV-infected patients with chronic periodontitis. *Journal of Periodontology*, *90*2, 167–176. https://doi.org/10.1002/JPER.18-0197

Noguera-Julian, M., Guillén, Y., Peterson, J., Reznik, D., Harris, E. V., Joseph, S. J., Rivera, J., Kannanganat, S., Amara, R., Nguyen, M. L., Mutembo, S., Paredes, R., Read, T. D., & Marconi, V. C. 2017. Oral microbiome in HIV-associated periodontitis. *Medicine*, *96*12, e5821. https://doi.org/10.1097/MD.0000000000005821

Noro Filho, G. A., Casarin, R. C. V., Casati, M. Z., & Giovani, E. M. 2012. PDT in non-surgical treatment of periodontitis in HIV patients: A split-mouth, randomized clinical trial. *Lasers in Surgery and Medicine*, *44*4. https://doi.org/10.1002/lsm.22016

Noro Filho, G. A., Salgado, D. M. R. D. A., Casarin, R. C. V., Casati, M. Z., Costa, C., & Giovani, E. M. 2013. Anti-infective periodontal therapy promoting improvement in systemic markers of HIV infection. *AIDS Research and Human Retroviruses*, *29*7. https://doi.org/10.1089/aid.2012.0359

Nouaman, M. N., Meless, D. G., Coffie, P. A., Arrivé, E., Tchounga, B. K., Ekouévi, D. K., Anoma, C., Eholié, S. P., Dabis, F., & Jaquet, A. 2015. Oral health and HIV infection among female sex workers in Abidjan, Côte d’Ivoire. *BMC Oral Health*, *15*1. https://doi.org/10.1186/s12903-015-0129-0

Odden, K., Schenck, K., Koppang, H., & Hurlen, B. 1994. Candidal infection of the gingiva in HIV‐infected persons. *Journal of Oral Pathology & Medicine*, *23*4. https://doi.org/10.1111/j.1600-0714.1994.tb01109.x

Pavan, P., Pereira, V. T., Souza, R. C., Souza, C. O., Torres, S. R., Colombo, A. P. V., Da Costa, L. J., Sansone, C., Deuzeda, M., & Gonçalves, L. S. 2014. Levels of HIV-1 in subgingival biofilm of HIV-infected patients. *Journal of Clinical Periodontology*, *41*11. https://doi.org/10.1111/jcpe.12306

Peppes, C. P., Lemos, A. S. P., Araujo, R. L. F., Portugal, M. E. G., Da Cruz Magalhães Buffon, M., & Raboni, S. M. 2013. Oral lesions frequency in HIV-positive patients at a tertiary hospital, Southern Brazil. *Brazilian Journal of Oral Sciences*, *12*3. https://doi.org/10.1590/S1677-32252013000300012

Persson, R. E., Hollender, L. G., & Persson, G. R. 1998. Alveolar Bone Levels in AIDS and HIV Seropositive Patients and in Control Subjects. *Journal of Periodontology*, *69*9. https://doi.org/10.1902/jop.1998.69.9.1056

Price, P., Calder, D. M., Witt, C. S., Allcock, R. J. N., Christiansen, F. T., Davies, G. R., Cameron, P. U., Rogers, M., Baluchova, K., Moore, C. B., & French, M. A. 1999. Periodontal attachment loss in HIV-infected patients is associated with the major histocompatibility complex 8.1 haplotype HLA-A1,B8,DR3. *Tissue Antigens*, *54*4. https://doi.org/10.1034/j.1399-0039.1999.540409.x

Rai, S., Subramanyam, G., Kumar, G., & Bhushan, V. 2022. Assessment of oral mucosal lesions among HIV positive transgenders residing in Odisha with and without Antiretroviral therapy. *Journal of Family Medicine and Primary Care*, *11*11, 7106. https://doi.org/10.4103/jfmpc.jfmpc_940_22

Ranganathan, A. T., Saraswathi, P. K., Albert, V., Baba, M. G. K., & Panishankar, K. H. 2012. Route of transmission might influence the clinical expression of periodontal lesions in “human immunodeficiency virus” positive patients. *Nigerian Journal of Clinical Practice*, *15*3. https://doi.org/10.4103/1119-3077.100646

Ranganathan, K., Magesh, K. T., Kumarasamy, N., Solomon, S., Viswanathan, R., & Johnson, N. W. 2007. Greater severity and extent of periodontal breakdown in 136 south Indian human immunodeficiency virus seropositive patients than in normal controls: A comparative study using community periodontal index of treatment needs. *Indian Journal of Dental Research*, *18*2. https://doi.org/10.4103/0970-9290.32420

Riley, C., London, J. P., & Burmeister, J. A. 1992. Periodontal health in 200 HIV‐positive patients. *Journal of Oral Pathology & Medicine*, *21*3. https://doi.org/10.1111/j.1600-0714.1992.tb00995.x

Ríos, C. N., Díaz, M. E. G., Rodríguez, V. Y. L., Moreno, V. H., & Romero, L. P. 2002. Enfermedad periodontal en pacientes infectados por el VIH. *Revista Cubana de Estomatologia*, *39*1.

Rozra, S., Kundu, D., Saha, B., Rudra, A., Chakrabarty, S., & Bharati, P. 2012. Periodontal status of HIV infected patients with special reference to CD4 cell count in West Bengal, India. *Asian Pacific Journal of Tropical Disease*, *2*6. https://doi.org/10.1016/S2222-18081260102-9

Sampath, C., Harris, E. P., Berthaud, V., Tabatabai, M. A., Wilus, D. M., Crayton, M. A., Moss, K., Webster-Cyriaque, J., Southerland, J. H., Koethe, J. R., & Gangula, P. R. 2022. Periodontal Treatment Reduces Circulating Pro-Inflammatory Cytokine and Chemokine Levels in African American HIV+ Individuals with Virological Suppression. *Journal of Dental Applications*, *8*1, 477–487.

Santo, A. E., Tagliaferro, E. P. S., Ambrosano, G. M. B., Meneghim, M. C., & Pereira, A. C. 2010. Dental status of Portuguese HIV+ patients and related variables: A multivariate analysis. *Oral Diseases*, *16*2. https://doi.org/10.1111/j.1601-0825.2009.01622.x

Schuettfort, G., de Leuw, P., Haberl, A., Herrmann, E., Park, K. H., Wolf, T., & Stephan, C. 2018. HLA-b57.01 shields people living with HIV for significantly better periodontal health. *Journal of Periodontology*, *89*8. https://doi.org/10.1002/JPER.17-0532

Scully, C., Porter, S. R., Mutlu, S., Epstein, J. B., Glover, S., & Kumar, N. 1999. Periodontopathic bacteria in English HIV-seropositive persons. *AIDS Patient Care and STDs*, *13*6. https://doi.org/10.1089/apc.1999.13.369

Sehdev, B., Tobar, M., Singh, R., Prasanna Kumar, P., & Sridhar, B. 2019. EVALUATION OF PERIODONTAL STATUS OF HIV INFECTED PATIENTS WITH SPECIAL REFERENCE TO CD4 CELL COUNT IN MEKELLE HOSPITAL, ETHIOPIA. *International Journal of Pharmaceutical, Chemical & Biological Sciences*, *9*1, 42–46. www.ijpcbs.com

Shaghaghian, S., Homayooni, M., Amin, M., & Rezazadeh, F. 2021. Oral health status of patients infected with human immunodeficiency virus and related factors, Iran: a cross-sectional study. *BMC Oral Health*, *21*1, 657. https://doi.org/10.1186/s12903-021-02002-3

Shugars, D. C., Slade, G. D., Patton, L. L., & Fiscus, S. A. 2000. Oral and systemic factors associated with increased levels of human immunodeficiency virus type 1 RNA in saliva. *Oral Surgery, Oral Medicine, Oral Pathology, Oral Radiology, and Endodontics*, *89*4. https://doi.org/10.1016/S1079-21040070124-7

Teanpaisan, R., Douglas, C. W. I., & Nittayananta, W. 2001. Isolation and genotyping of black-pigmented anaerobes from periodontal sites of HIV-positive and non-infected subjects in Thailand. *Journal of Clinical Periodontology*, *28*4. https://doi.org/10.1034/j.1600-051x.2001.028004311.x

Tomar, S. L., Swango, P. A., Kleinman, D. V., & Burt, B. A. 1995. Loss of Periodontal Attachment in HIV-Seropositive Military Personnel. *Journal of Periodontology*, *66*6. https://doi.org/10.1902/jop.1995.66.6.421

Trentin, M. S., Scortegagna, S. A., Dalbello, M. S., Bittencourt, M. E. de, Linden, M. S. S., Argenta, S., Casalli, M., Neves, M., Carli, J. P. De, & Oliveira, C. A. de. 2007. Periodontal disease and risk factors in HIV positive patients. *Rev Fac Odontol UPF*, *12*3, 49–55.

Tukutuku, K., Muyembe‐Tamfum, L., Kayembe, K., Mavuemba, T., Sangua, N., & Sekeie, I. 1990. Prevalence of dental caries, gingivitis, and oral hygiene in hospitalized AIDS cases in Kinshasa, Zaire. *Journal of Oral Pathology & Medicine*, *19*6. https://doi.org/10.1111/j.1600-0714.1990.tb00840.x

Umeizudike, K. A., Ayanbadejo, P. O., Savage, K. O., Akanmu, A. S., Nwhator, S. O., & Emeka, C. I. 2014. Prevalence and Determinants of Chronic periodontitis in HIV positive patients in Nigeria. *Asian Pacific Journal of Tropical Disease*, *4*4. https://doi.org/10.1016/S2222-18081460578-8

Vastardis, S. A., Yukna, R. A., Fidel, P. L., Leigh, J. E., & Mercante, D. E. 2003. Periodontal Disease in HIV-Positive Individuals: Association of Periodontal Indices with Stages of HIV Disease. *Journal of Periodontology*, *74*9. https://doi.org/10.1902/jop.2003.74.9.1336

Vernon, L. T., Demko, C. A., Babineau, D. C., Wang, X., Toossi, Z., Weinberg, A., & Rodriguez, B. 2013. Effect of Nadir CD4+ T Cell Count on Clinical Measures of Periodontal Disease in HIV+ Adults before and during Immune Reconstitution on HAART. *PLoS ONE*, *8*10. https://doi.org/10.1371/journal.pone.0076986

Vernon, L. T., Seacat, J. D., Demko, C. A., Paes B. da Silva, A., & Zyzanski, S. J. 2019. A provider-observed tool to assess Oral Hygiene Skill Mastery OHSIM in human immunodeficiency virus-positive HIV+ adults. *Special Care in Dentistry*, *39*2. https://doi.org/10.1111/scd.12355

Williams‐Wiles, L., & Vieira, A. R. 2019. HIV status does not worsen oral health outcomes. *Journal of Clinical Periodontology*, *46*6, 640–641. https://doi.org/10.1111/jcpe.13116

Wulandari, E. A. T., Wijaya, I. P., Karim, B., Ariyanto, I., Tanudjaja, S. A., Lee, S., & Price, P. 2020. periodontitis and cytomegalovirus associate with atherosclerosis among HIV patients after 5 years on ART. *JAIDS Journal of Acquired Immune Deficiency Syndromes*, *85*2, 195–200. https://doi.org/10.1097/QAI.0000000000002417

Yeung, S. C. H., Stewart, G. J., Cooper, D. A., & Sindhusake, D. 1993. Progression of Periodontal Disease in HIV Seropositive Patients. *Journal of Periodontology*, *64*7. https://doi.org/10.1902/jop.1993.64.7.651

Yeung, S. C. H., Taylor, B. A., Sherson, W., Lazarus, R., Zhao, Z. Z., Bird, P. S., Hamlet, S. M., Bannon, M., Daly, C., & Seymour, G. J. 2002. IgG Subclass Specific Antibody Response to Periodontopathic Organisms in HIV-Positive Patients. *Journal of Periodontology*, *73*12. https://doi.org/10.1902/jop.2002.73.12.1444
